# Preclinical spheroid models identify *BMX* as a therapeutic target for metastatic MYCN nonamplified neuroblastoma

**DOI:** 10.1172/jci.insight.169647

**Published:** 2024-07-22

**Authors:** Santhoshkumar Sundaramoorthy, Daniele Filippo Colombo, Rajendran Sanalkumar, Liliane Broye, Katia Balmas Bourloud, Gaylor Boulay, Luisa Cironi, Ivan Stamenkovic, Raffaele Renella, Fabien Kuttler, Gerardo Turcatti, Miguel N. Rivera, Annick Mühlethaler-Mottet, Anaïs Flore Bardet, Nicolò Riggi

**Affiliations:** 1 Experimental Pathology Service, Lausanne University Hospital and University of Lausanne, Lausanne, Switzerland.; 2 Infectious Diseases Biomarkers, Janssen Research and Development, Beerse, Belgium.; 3 Department Woman-Mother-Child, Division of Pediatrics, Lausanne University Hospital and University of Lausanne, Lausanne, Switzerland.; 4 Department of Pathology and Cancer Center, Massachusetts General Hospital and Harvard Medical School.; 5 Biomolecular Screening Facility, Swiss Federal Institute of Technology (EPFL), Lausanne, Switzerland.; 6 Biotechnology and Cell Signaling (BSC), CNRS UMR7242, University of Strasbourg, Illkirch, France.; 7 Institute of Genetics and Molecular and Cellular Biology (IGBMC), CNRS UMR7104, University of Strasbourg, INSERM U1258, Illkirch, France.

**Keywords:** Therapeutics, Cancer, Epigenetics, Oncogenes

## Abstract

The development of targeted therapies offers new hope for patients affected by incurable cancer. However, multiple challenges persist, notably in controlling tumor cell plasticity in patients with refractory and metastatic illness. Neuroblastoma (NB) is an aggressive pediatric malignancy originating from defective differentiation of neural crest–derived progenitors with oncogenic activity due to genetic and epigenetic alterations and remains a clinical challenge for high-risk patients. To identify critical genes driving NB aggressiveness, we performed combined chromatin and transcriptome analyses on matched patient-derived xenografts (PDXs), spheroids, and differentiated adherent cultures derived from metastatic MYCN nonamplified tumors. Bone marrow kinase on chromosome X (BMX) was identified among the most differentially regulated genes in PDXs and spheroids versus adherent models. BMX expression correlated with high tumor stage and poor patient survival and was crucial to the maintenance of the self-renewal and tumorigenic potential of NB spheroids. Moreover, BMX expression positively correlated with the mesenchymal NB cell phenotype, previously associated with increased chemoresistance. Finally, BMX inhibitors readily reversed this cellular state, increased the sensitivity of NB spheroids toward chemotherapy, and partially reduced tumor growth in a preclinical NB model. Altogether, our study identifies BMX as a promising innovative therapeutic target for patients with high-risk MYCN nonamplified NB.

## Abstract 2

Comparison of neuroblastoma models identifies BMX as a marker of aggressive forms of the disease, suggesting potential as a therapeutic target.

## Introduction

Tumor cell identity is the product of complex and dynamic interactions between oncogenic events and mechanisms regulating normal differentiation pathways, resulting in intratumor hierarchies governing tumor-initiating potential and response to therapy (1–4). Attempts to increase therapeutic effectiveness in malignancies with documented cellular hierarchies should, therefore, include strategies targeting not only rapidly dividing lineage-committed tumor cells but also targeting slowly dividing and resistant immature cells. This notion is particularly relevant in pediatric cancers, where a limited number of selected genetic alterations prevent normal lineage trajectories, enhancing cellular proliferation and transformation (5, 6). Therapeutic approaches aimed at targeting specific cellular states or prompting differentiation may therefore represent promising clinical options for these diseases.

Neuroblastoma (NB) is a pediatric cancer of the developing sympathetic nervous system and one of the most common solid tumors in infancy. A hallmark of NB is its major heterogeneity, reflected in clinical outcomes ranging from spontaneous regression to extreme malignancy and, in the diversity of tumor cell phenotypes, encompassing both primitive and well-differentiated tumor cells. Established risk factors include older age (>18 months) at diagnosis, higher stages, and undifferentiated histological features, with approximatively half of diagnosed NB belonging to the high-risk (HR) group associated with poor survival (7–10). Among the best characterized genetic alterations that underlies NB development and progression are *MYCN* amplification, segmental chromosomal aberrations, *ALK* amplification/activating mutations, *TERT* rearrangements, *ATRX* deletions, and alterations in TP53 or RAS/MAPK pathways (11–15). Additional recurrent genetic alterations are rarely identified in NB and involve chromatin remodelers, such as *ARID1A/B* (16), indicating that additional changes in epigenetic regulatory mechanisms may be involved in the pathogenesis of these tumors. In keeping with this, multiple studies have reported epigenetic perturbations leading to abnormal expression of genes involved in development, differentiation, proliferation, and apoptosis to support the malignant phenotype of NB tumor cells (17, 18).

The marked heterogeneity displayed by NB tumors is further highlighted by the presence of intratumor cellular hierarchies and divergent cellular states that rely on distinct epigenetic circuitries. In line with this, chromatin and transcriptional profiling of NB tumor cells have recently identified 2 phenotypes that represent divergent differentiation states, defined as committed adrenergic/noradrenergic (NOR) and uncommitted mesenchymal (MES)/neural crest-like cells (19, 20). These 2 identities are supported by the expression of distinct lineage-specific transcription factors (TFs) associated with superenhancers (SEs), and assembled into specific core regulatory circuitries (CRCs) that enable tumor cells to undergo spontaneous and bidirectional interconversion by epigenetic reprogramming (19, 20). Importantly, MES-like cells are rare in primary NB tumors and display multiple defining features of cancer stem cells. These include a low proliferation rate, high lineage plasticity, enhanced migratory properties, and a transcriptional program close to Schwann Cell Precursors (21). Consistent with this, the MES phenotype has been associated with increased tumor relapse and therapeutic resistance, including the ability of NOR-to-MES reprogrammed cells to escape from ALK targeted therapies (22).

Collectively, these observations support cell plasticity as a major mechanism of drug resistance, tumor dissemination, and relapse in NB (23), and they highlight the need to design cell state–specific therapies preventing cellular reprogramming. Due to the heterogeneous nature of NB tumors, however, identifying druggable targets has remained a challenging endeavor. This requires the generation of adequate preclinical models that faithfully recapitulate tumor heterogeneity and can be interrogated to nominate new and cell state-specific druggable targets. In keeping with this, recent developments in spheroid/organoid culture methods have enabled researchers to better model tumor heterogeneity in vitro, to an extent allowing the identification of drug targets that remained otherwise elusive in conventional 2D culture methods (24–30). Similarly, NB organoids established from patient-derived tumor tissues have been shown to display cellular heterogeneity and to retain an increased tumorigenic potential as compared with their 2D culture counterparts, offering a model of choice to interrogate the molecular underpinnings of their malignant phenotype (31).

Here we leveraged our expertise in NB modeling to investigate the epigenetic and transcriptional landscape of matched spheroid and adherent NB culture models, generated from patient-derived xenografts (PDX) of *MYCN* nonamplified (nMNA) aggressive primary bone marrow metastases. The comparative RNA-Seq and H3K4me3 analysis of tumor xenografts, spheroids, and adherent cultures led to the identification of a 12-gene signature with elevated expression in tumors and spheroid models as compared with adherent cells. Among these candidates, we focused on the bone marrow kinase on chromosome X gene (*BMX*), whose expression increased from NB stage 1 to 4, and was associated with poor prognosis in multiple data sets of patients with nMNA NB. We identified a hitherto unrecognized role for BMX in sustaining the self-renewal and tumorigenic properties of NB spheroid cultures, at least partially through its role in supporting their mesenchymal phenotype. Altogether, our work provides an unprecedented view of the regulatory landscape of primary preclinical NB models, and it delineates a druggable and cell state–specific therapeutic target for the most advanced and lethal stage of this disease.

## Results

### Matched transcriptional and chromatin H3K4me3 profiling of primary nMNA NB models identifies BMX as a marker of tumor xenografts and spheroids.

**Figure 1:**
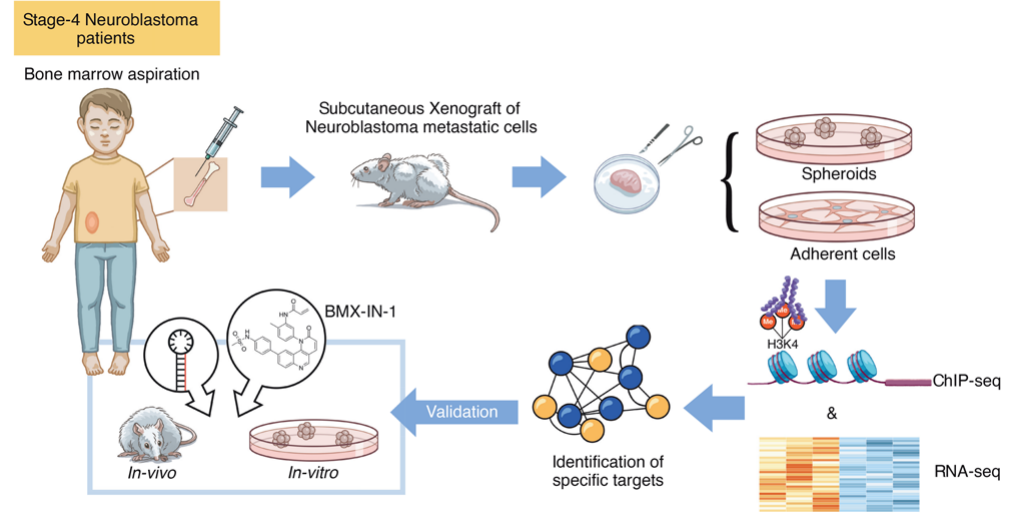
Schematic diagram depicting the establishment and profiling of preclinical NB models. Metastatic NB cells were collected from bone marrow aspirates of patients with stage 4 HR-NB and were maintained in vivo by serial s.c. transplantations into Swiss nude mice. Tumor xenografts were resected when reaching the authorized volume, and single-cell suspension were prepared to generate matched spheroid and adherent cell culture models. Both cellular models and their xenografts of origin were profiled by RNA-Seq and ChIP-Seq, and genes found to be preferentially induced in xenografts and spheroids were selected for further functional validation in vitro and in vivo.

Multiple studies (including from our own group) have shown that different culture conditions successfully enrich primary NB cultures for selected tumor phenotypes. Patient-derived NB cells grown as spheroids in serum-free conditions show distinctive biological features as compared with their differentiated counterparts, consistent with a modulation of the stemness-differentiation axis by serum (31, 32). Importantly, the alternate culture conditions confer NB cells with distinct functional properties, including differential stem cell marker expression, drug resistance, and in vivo tumor-initiating potential (31, 33). To identify distinguishing features of aggressive NB cells, we expanded matched pairs of NB cultures derived from 2 different bone marrow aspirates either as spheroids grown in serum-free conditions or as NB cells grown as adherent monolayers in serum (Figure 1 and Supplemental Table 1; supplemental material available online with this article; https://doi.org/10.1172/jci.insight.169647DS1) . Given that deregulated *MYCN* expression is known to dominate the active *cis*-regulatory landscape of *MYCN*-amplified (MNA) NB tumors (34), we reasoned that focusing on nMNA tumors, which still represent approximatively 50% of HR-NB, may provide us with a better opportunity to identify additional oncogenic factors promoting the most aggressive state of this disease. Due to the limited amount of material provided by bone marrow aspirates, metastatic NB cells were initially expanded as xenografts into immunocompromised mice and maintained in vivo by serial xeno-transplantations prior to in vitro culture (Figure 1) (31).

**Figure 2:**
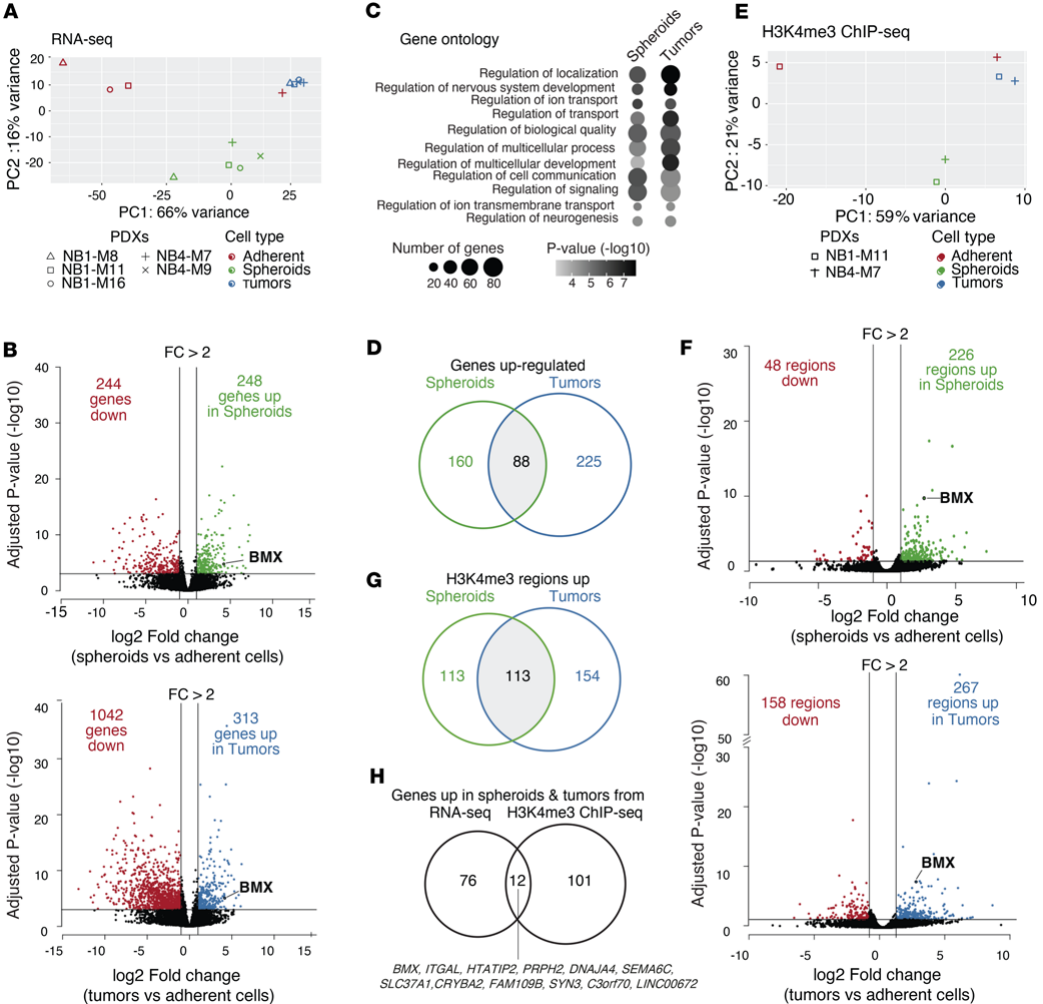
Matched transcriptional and chromatin H3K4me3 profiling of primary nMNA NB models identifies BMX as a marker of tumor xenografts and spheroids. (**A**) Principal component analysis (PCA) of RNA-Seq data showing clustering of the samples according to cell types (adherent, spheroids or tumors). (**B**) Volcano plots showing differentially expressed genes in spheroids vs. adherent samples (top) or tumor vs. adherent samples (bottom). Genes considered as significantly upregulated or downregulated (DEseq2 fold change > 2 and adjusted *P* < 0.001) are highlighted in green (top) or blue (bottom) and red, respectively. (**C**) Bubble plot representing the enrichment of gene ontology biological processes in genes significantly enriched in spheroids vs. adherent samples (left) or tumors vs. adherent samples (right) and their associated hypergeometric *P* value. (**D**) Venn diagrams showing the overlap (gray) of genes upregulated in spheroids vs. adherent samples (left; green) or tumors vs. adherent samples (right; blue). (**E**) Principal component analysis (PCA) of the H3K4me3 ChIP-Seq data showing clustering of the samples according to cell type (adherent, spheroids, or tumors). (**F**) Volcano plots showing differentially enriched H3K4me3 regions in spheroids vs. adherent samples (top) or tumors vs. adherent samples (bottom). TSS regions significantly upregulated or downregulated (DEseq2 fold change > 2 and adjusted *P* < 0.05) are highlighted in green (top) or blue (bottom) and red, respectively. (**G**) Venn diagram showing the overlap (gray) of H3K4me3 regions enriched in spheroids vs. adherent samples (left; green) or tumors vs. adherent samples (right; blue). (**H**) Venn diagram showing the overlap of genes upregulated in both spheroids and tumors (88 from overlap in **D**) and genes of which the TSS is closest to the H3K4me3 regions enriched in both spheroids and tumors (113 from overlap in **G**).

Previous studies have shown the power of combining chromatin and transcriptional analyses to delineate key determinants of cell states in normal development and cancer (35, 36), enabling the identification of genes playing major roles in maintaining cell identities. Based on these observations, we set out to define potential epigenetic determinants of NB malignancy by generating matched H3K4me3 ChIP-Seq profiles (associated with active promoters) and RNA-Seq transcriptional landscapes of NB spheroids and then comparing them against their differentiated counterparts. Principal component analysis (PCA) on the transcriptional profiles for the NB1 and NB4 tumors grown either as spheroids or adherent cultures revealed that the culture models tend to cluster together, with the exception of NB4-M7 adherent cells, therefore showing distinct expression programs (Figure 2A). For each tumor model, we also profiled the corresponding xenograft of origin, which displayed a separate clustering. We then set out to define differential gene expression patterns between tumors, spheroids, and their matched differentiated adherent counterparts in both tumor models. Using a fold change > 2 and an adjusted *P* < 1 × 10^–3^, this analysis revealed 248 and 244 genes to be, respectively, induced and repressed in NB spheroids compared with adherent NB cells (Figure 2B and Supplemental Table 2). When the same analysis was repeated comparing matched NB tumors and adherent cells, we identified 313 and 1,042 genes to be induced and repressed in tumors, respectively, compared with adherent NB cells (Figure 2B and Supplemental Table 2). The functional annotation (gene ontology biological processes) of the genes showing higher expression in spheroids or tumors compared with adherent models showed a similar enrichment for terms related to the regulation of nervous system development and neurogenesis, consistent with the divergent differentiation states of the tumor models (Figure 2C and Supplemental Table 2). Interestingly, when the 248 genes upregulated in NB spheroids were compared with the 313 genes upregulated in NB tumors, we observed a substantial overlap of 88 genes, which represents transcripts enriched both in NB stem-like models and in their tumor of origin relative to the adherent cells (Figure 2D).

Next, in order to prioritize our list of differentially expressed genes for transcripts with potential regulatory roles in the spheroid cultures, we performed similar analyses on matched H3K4me3 ChIP-Seq profiles. As for the transcriptional profiles, PCA of H3K4me3 signals also showed that NB tumors, spheroids, and adherent cultures tend to cluster based on their status, with the exception of NB4-M7 adherent cells (Figure 2E). Differential analysis of H3K4me3 signals at promoters of these 3 tumor models identified sets of transcriptional start sites (TSSs) with higher H3K4me3 signal in spheroids and tumors, as compared with adherent cultures (fold change > 2 and an adjusted *P* < 0.05; Figure 2F and Supplemental Table 3). In particular, 113 TSSs were found to be enriched for the H3K4me3 mark in both tumor and spheroid models, as compared with matched differentiated cultures (Figure 2G). In order to refine the list of candidate targets, we therefore reasoned that genes playing important roles in maintaining the most aggressive cellular phenotype in metastatic NB tumors should show higher expression in tumor and spheroid models, while displaying markedly differences in H3K4me3 signal at their TSSs, testifying for their developmental relevance. Integrated transcriptional and chromatin comparative analysis revealed a 12-gene signature that shows high expression level and promoter activity in both NB tumors and spheroids (Figure 2H). Among these genes, *BMX* caught our attention because of its reported role in maintaining glioblastoma stem cells and the possibility to pharmacologically target its enzymatic function (37, 38).

### Elevated BMX expression is associated with advanced clinical stage and poor prognosis in patients with nMNA NB.

**Figure 3:**
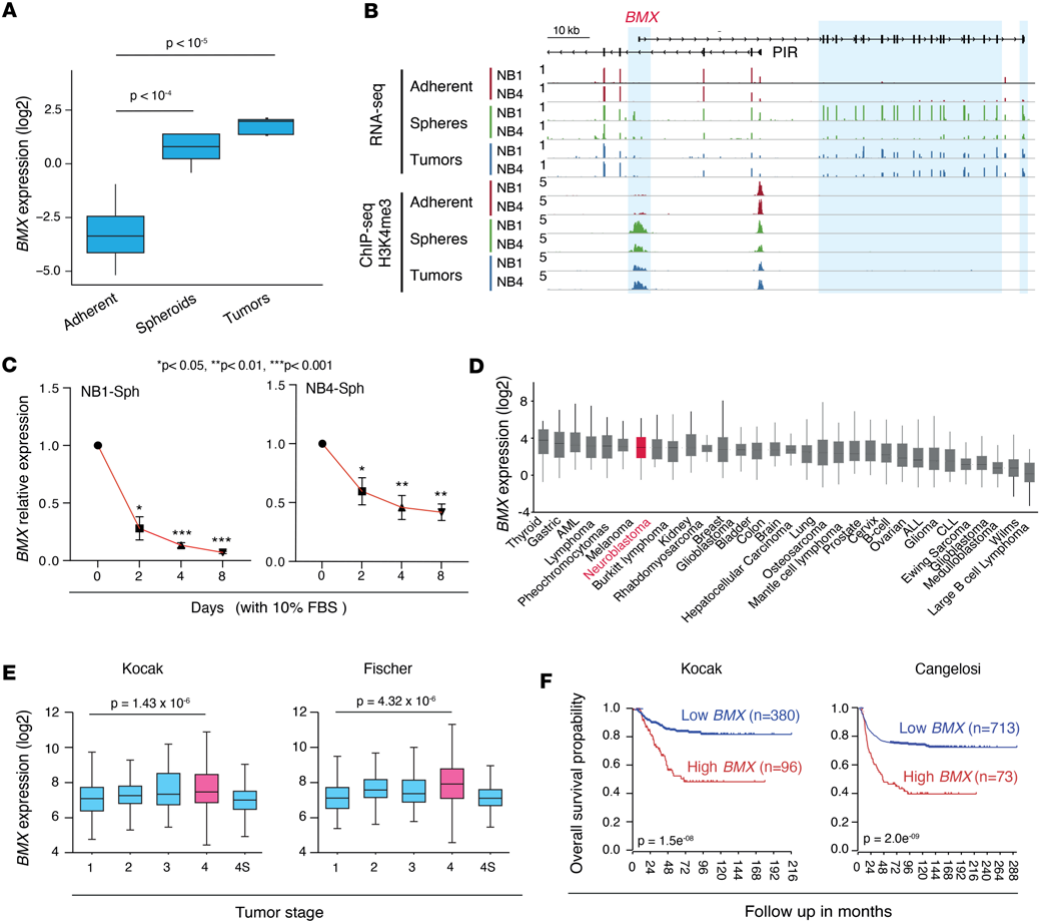
High *BMX* expression levels correlate with advanced stage and poor prognosis in nMNA neuroblastoma patients. (**A**) Box plot showing *BMX* expression from the RNA-Seq data in NB adherent cells, spheroids, and tumors. Adjusted *P* values from the DEseq2 analysis. (**B**) Genome browser tracks of RNA-Seq and H3K4me3 ChIP-Seq signals for NB1-M11 and NB4-M7 models at the *BMX* genomic locus. Blue zones mark the TSS (left) and exons (right) of the BMX gene. (**C**) qPCR assessment of *BMX* transcripts after differentiation of NB1 (left) and NB4 (right) spheroids by serum (10% FBS) at indicated times. Relative expression as mean ± SEM values of 3 technical replicates are shown. Statistical analysis was done by unpaired *t* test. (**D**) *BMX* mRNA expression levels across 31 tumor types obtained from the R2 genomics platform. (**E**) BMX mRNA expression levels in primary NB samples included in the Kocak and Fischer NB cohorts from the R2 genomics platform. Patient samples were grouped based on International Neuroblastoma Staging System (INSS) stages 1 to 4s, and Welch *P* value is shown. (**F**) Kaplan-Meier survival curves of patients with NB included in the Kocak and Cangelosi data sets from the R2 genomics platform stratified by *BMX* expression levels. Statistical analyses were performed using the log-rank test. Data shown in **D**–**F** are from publicly available patient cohorts (R2: visualization platform). *P < 0.05, **P < 0.01, ***P < 0.001.

Consistent with our analyses, *BMX* mRNA expression levels were increased in NB tumor and spheroid models (Figure 3A and Supplemental Figure 1A), and the gene TSS showed higher H3K4me3 deposition when compared with adherent cultures (Figure 3B). As an orthogonal approach, we also performed RNA-ISH and confirmed the expression of *BMX* mRNA in both NB1- and NB4-derived xenografts (Supplemental Figure 1B). To confirm that differences in *BMX* expression reflect global changes in cellular differentiation and are not related to potential genetic alterations or positive selection events occurring during the establishment of our culture system, NB1 and NB4 spheroids were cultured in the presence of serum for a short time period of 8 days, during which *BMX* expression was monitored. For both spheroid models, serum addition resulted in rapid cell adherence alongside a progressive decrease in *BMX* expression (Figure 3C and Supplemental Figure 1C), consistent with the notion that the expression of this kinase is restricted to a less-differentiated tumor cell phenotype, as previously reported in glioblastoma (37).

Subsequently, to correlate our preclinical models to the patient reality, we surveyed *BMX* expression across a panel of 31 tumor types using transcriptomic data sets from the R2 Genomics Analysis and Visualization Platform (r2.amc.nl). Although global *BMX* transcript levels remained relatively low across all tumor types analyzed, as expected for a marker of undifferentiated tumor subpopulations, NB tumors ranked seventh in terms of *BMX* expression (Figure 3D). To further validate the clinical relevance of our findings, we also interrogated 2 NB transcriptomic data sets for *BMX* expression levels across different tumor stages. *BMX* expression increased from stage 1 (localized) to 4 (metastatic) in both data sets, while it remained low in stage 4s, which represent the metastatic stage that spontaneously regress in infants younger than 18 months (Figure 3E). A similar consistent trend was not identified for the majority of the remaining 10 protein-coding genes that compose our initial signature (Supplemental Figure 1D). These results prompted us to determine if *BMX* expression levels bear a prognostic value for patients with NB. To this end, the overall survival of patients with NB from 2 different NB cohorts were stratified based on *BMX* expression levels. All data sets showed that high *BMX* levels correlate with strongly decreased overall survival for patients with NB (Figure 3F). Interestingly, when the same analysis was repeated after segregating patients with NB based on the presence or absence of *MYCN* amplification, we observed a similar trend for nMNA but not MNA tumors (Supplemental Figure 1E), a result that cannot be explained by differences in *BMX* expression between nMNA and MNA tumors (Supplemental Figure 1F) but rather suggest that *BMX* expression may be a selective clinical indicator for patients with NB lacking *MYCN* amplification.

### BMX expression is essential for the clonogenic and self-renewal abilities of NB spheroid models.

**Figure 4:**
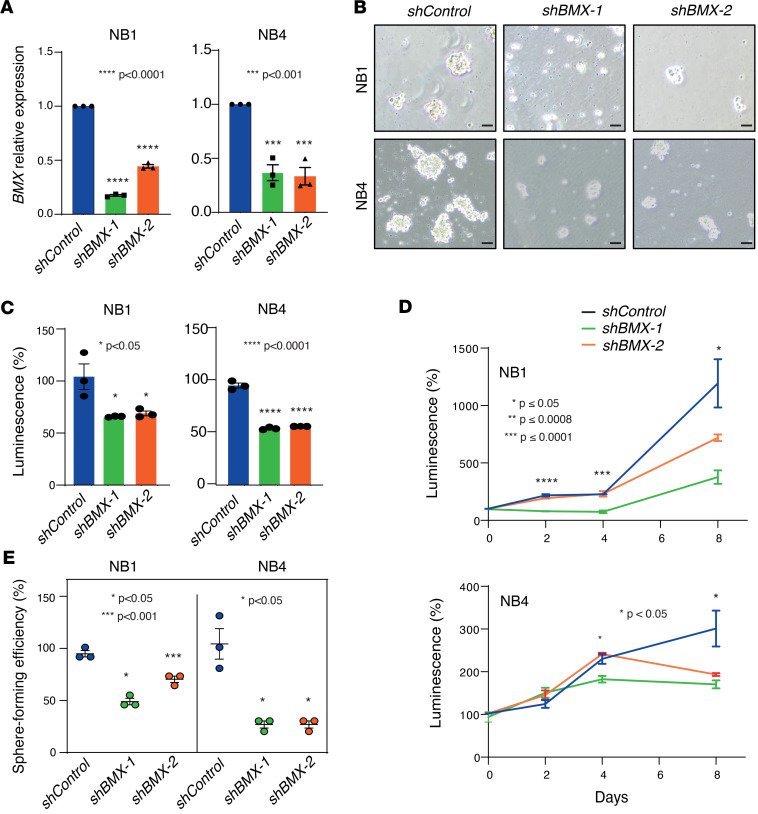
*BMX* expression is critical for NB spheroid clonogenicity and self-renewal. (**A**) qPCR showing relative *BMX* mRNA expression levels in NB1 (left) and NB4 (right) spheroids infected with lentivirus-carrying *BMX*-targeting shRNAs sequences (sh*BMX*#1 and sh*BMX*#2) compared with control spheroids transduced with a GFP-targeting shRNA (shControl). (**B**) Micrographs illustrating the changes in NB1 and NB4 spheroids morphology at day 4 after transduction with either shControl, sh*BMX*#1, or sh*BMX*#2 lentiviruses**.** Representative images are shown. Scale bars: 100 μm. Magnification, 10×. (**C**) Graph showing the viability of the same NB1 and NB4 spheroid models as in **B**, measured using the CellTiter-Glo Luminescent Cell Viability Assay kit. (**D**) Time-dependent analysis of spheroids viability measured as in **C**. (**E**) Graph depicting the changes in sphere forming ability of NB1 and NB4 FACS-sorted single cells transduced with either shControl, sh*BMX*#1, or sh*BMX*#2 lentiviruses. (**A** and **C**–**E**) Individual values and mean ± SEM values of 3 technical replicates are shown. (**A**, **C**, and **E**) Statistical analyses are performed using the 1-way ANOVA followed by post hoc pairwise comparisons to compute adjusted *P* values. (**D**) One-way ANOVA was used. *P ≤ 0.05, ***P < 0.001, ****P < 0.0001.

Next, we set out to interrogate the potential involvement of BMX in supporting the functional properties of our primary tumor models. To this end, we depleted *BMX* expression from both NB1 and NB4 spheroids using a shRNA-based lentiviral system. Both shRNA sequences efficiently reduced *BMX* mRNA expression in the 2 models, although not completely (Figure 4A), resulting in a marked decrease in their ability to form spheroids (Figure 4B). To better understand how a decrease in *BMX* expression results in reduced NB spheroids growth, we next evaluated potential changes in NB cellular viability, proliferation, and self-renewal following *BMX* depletion. First, assessment of NB1 and NB4 spheroid growth using an ATP-based luminescence reporter assay revealed that *BMX* knockdown leads to a decrease in spheroid viability (Figure 4C) and proliferation (Figure 4D). Second, given that NB spheroids have been previously reported to display self-renewal capabilities associated with sustained tumor initiation (31), we measured the capacity of individual cells to form new tumor spheres by a clonogenic assay, a widely used method to assess the effect of genetic and pharmacological perturbations on tumor cell self-renewal (37). For this purpose, we performed fluorescence-activated cell sorting (FACS) of individual viable tumor cells, followed by seeding them as single-cell clones in ultra-low–attachment 96-well plates and scoring for spheres formation after 30 days. Remarkably, *BMX* depletion led to a substantial reduction in the sphere-forming ability of both NB models, indicating a decrease in their self-renewal potential upon *BMX* depletion (Figure 4E). To extend these results to additional nMNA NB models, we generated xenografts with the nMNA NB cell line SK-N-AS in immunocompromised mice and derived similar spheroids cultures in which *BMX* expression was decreased by shRNA (Supplemental Figure 2A). Consistent with the results obtained in our primary tumor models, SK-N-AS spheroids also showed a marked decrease in viability, proliferation (Supplemental Figure 2B), and sphere-forming capacity (Supplemental Figure 2C) upon *BMX* depletion.

### nMNA NB spheroids display increased sensitivity toward BMX pharmacological inhibition.

**Figure 5:**
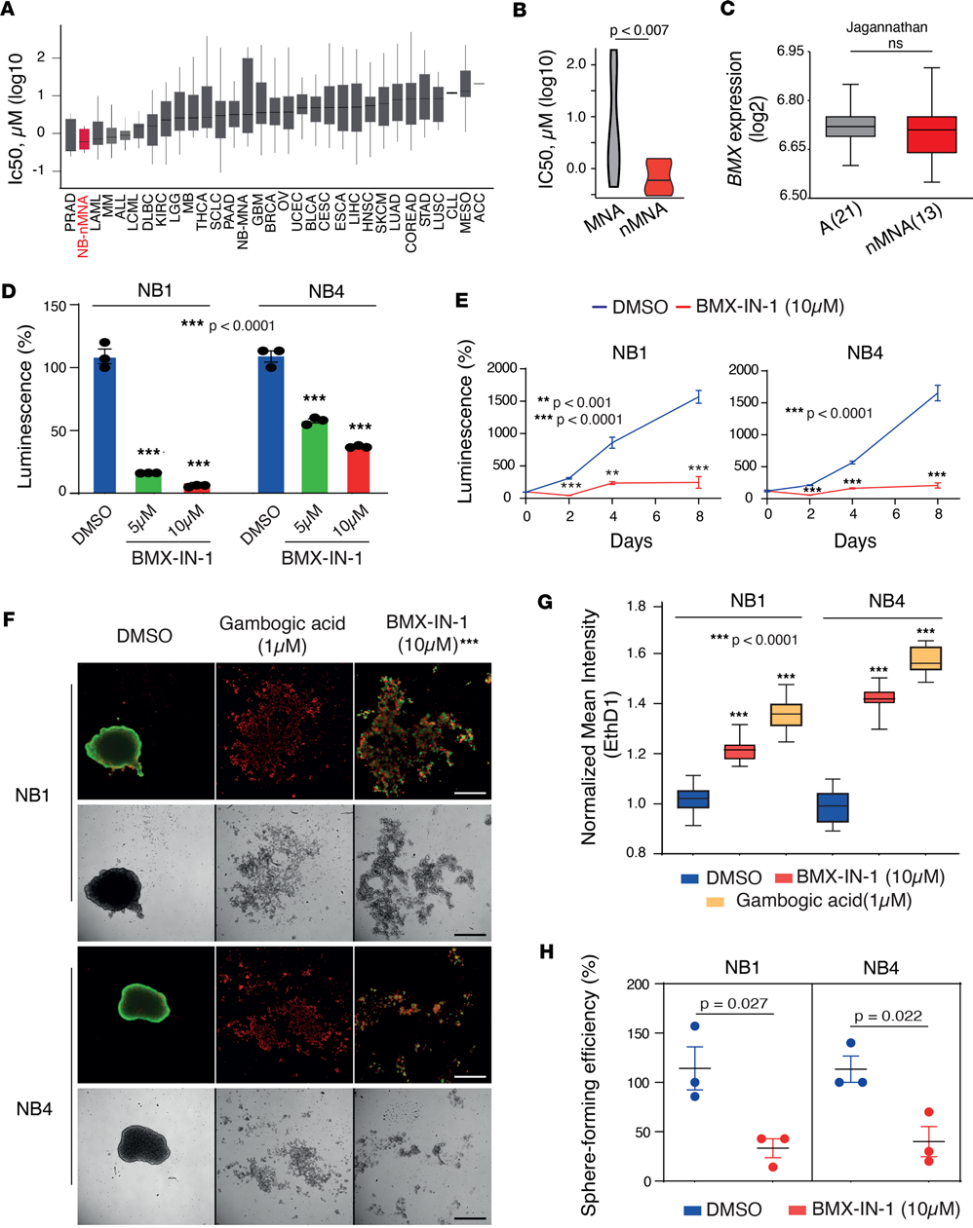
nMNA NB cell lines and primary spheroids are highly sensitive to BMX pharmacological inhibition. (**A**) Box plots depicting the sensitivity toward the *BMX* inhibitor QL-XII-47 for cancer cell lines across 31 different tumor types. Data were obtained from the Genomics of Drug Sensitivity in Cancer (GDSC) screening program and are presented as IC_50_ (μM). (**B**) Violin plots illustrating the differential sensitivity of NB cell lines toward the *BMX* inhibitor QL-XII-47 based on their *MYCN* amplification status. Data were obtained and presented as in **A**. (**C**) Box plots showing *BMX* mRNA expression levels for a panel of NB cell lines segregated based on their *MYCN* amplification status. The expression values of BMX were extracted from the Jagannathan data set available at R2 genomics platform. (**D**) Bar plots showing the viability of NB1 and NB4 spheroids treated with vehicle (DMSO) or BMX-IN-1 at 5 μM and 10 μM for 4 days. (**E**) Time-dependent analysis of spheroid viability as in **D**. (**F**) Representative images of NB1 (upper rows) and NB4 (lower rows) spheroids treated with DMSO as control, Gambogic acid (1 μM), and BMX-IN-1 (10 μM) for 7 days. Live cells were stained in green using Calcein AM, and dead cells in red using Ethidium homodimer-1. Objective, 4×/0.2. The entire well area was captured with 4 fields of view, and the representative images shown are cropped on the location of spheres in the wells. Scale bar: 1,000 μm. (**G**) Box plots showing the quantification of the assay shown in **F**, using normalized mean intensity of mean Ethidium homodimer-1 staining of spheroids treated with BMX-IN-1 (10 μM), DMSO, and Gambogic acid (1 μM). (**H**) Graph depicting the changes in sphere forming ability of NB1 and NB4 single cells sorted by FACS treated with 10 μM of BMX-IN-1 or vehicle (DMSO). (**D**–**F**, and **H**) Mean ± SEM values of 3 technical replicates are shown. Statistical analyses are performed using the unpaired *t* test (**E**, **F**, and **H**) and 1-way ANOVA followed by post hoc pairwise comparisons (**D** and **G**) to compute adjusted *P* values. **P < 0.001, ***P < 0.0001.

To increase the translation effect of our findings, we then sought to determine if the pharmacological inhibition of BMX activity could represent a valuable therapeutic approach in nMNA tumors. To this purpose, we took advantage from an open-source database of drug sensitivity across cell lines derived from 30 different tumor types (www.cancerxxgene.org) (39), which includes the BMX inhibitor QL-XII-47. Remarkably, nMNA NB cell lines ranked second across all tumor types in terms of lowest IC_50_ for QL-XII-47 (Figure 5A) and exhibited markedly higher sensitivity than MNA NB lines profiled in the same study (Figure 5, A and B) despite similar *BMX* expression levels (Figure 5C), suggesting a selective dependency of nMNA NB tumors. We therefore set out to test pharmacological BMX inhibition in our cellular models, using the readily available irreversible BMX inhibitor BMX-IN-1, which has demonstrated efficacy in prostate cancer lines (38). Consistent with the results obtained in our shRNA-mediated depletion experiments, BMX pharmacological inhibition by BMX-IN-1 resulted in a substantial reduction in spheroids viability (Figure 5D and Supplemental Figure 2D) and proliferation (Figure 5E) for both NB models. The same held true when spheroids derived from the SK-N-AS xenografts were treated with similar concentrations of the BMX-IN-1 inhibitor (Supplemental Figure 2, D and E). To further substantiate our observations, we performed an image-based assay on both NB1 and NB4 models treated either with BMX-IN-1 or Gambogic acid (GA), a well-defined proapoptotic agent. As assessed by the relative fluorescence intensity of Calcein AM and Ethidium homodimer-1 staining, BMX inhibition resulted in a marked disassembly of the spheroids morphology (Figure 5F) and a cell death level close to GA, as compared with DMSO vehicle alone (Figure 5G). Moreover, similarly to BMX depletion, BMX-IN-1 also significantly decreased the sphere-forming potential of both NB1 and NB4 models (Figure 5H).

A possible challenge with pharmacological agents is their potential lack of specificity and/or presence of off-target effects. In order to assess the specificity of the BMX-IN-1 inhibitor, we applied similar experimental conditions to primary spheroid cultures derived from 2 other pediatric tumors, Ewing and Synovial sarcoma, which displayed very low *BMX* expression levels (Supplemental Figure 2F). As expected for a BMX-dependent effect in our NB models, neither sarcoma spheroid models showed changes in cell viability and sphere size upon BMX inhibition (Supplemental Figure 2, G and H). Consistent with this, BMX-IN-1 also failed to induce any change in the viability of matched adherent cells derived from NB1 and NB4 models, which display only minimal BMX expression levels (Supplemental Figure 1A and Supplemental Figure 2, I and J). Next, to exclude potential off-target effects of BMX-IN-1 in NB spheroids, we surveyed the RNA-Seq data from both spheroids and adherent cell populations for the expression levels of other kinases that are potentially also targeted by BMX-IN-1, although with much lower potency (40), such as BTK, BLK, JAK3, EGFR, ITK, and TEC. All these kinases displayed reduced expression levels, as compared with BMX, in both NB spheroid models. Furthermore, they showed similar or higher expression in the adherent NB models, which are resistant to BMX inhibition, suggesting that the biological effect observed in our spheroid models are specifically mediated by BMX inhibition (Supplemental Figure 2K). Finally, to gain better insight into the potential downstream pathways regulated by BMX in NB spheroids, we evaluated changes in phosphorylation for STAT3, since this protein has been reported as a downstream target of BMX in GBM stem cells (37) and is known to play key roles in normal and cancer stem cell maintenance (41, 42). Consistent with the findings reported in GBM stem cells, Western blot analysis for STAT3 phosphorylation levels (pSTAT3-Tyr705) in either control or BMX-IN-1–treated NB1 and NB4 spheroids confirmed a marked reduction in pSTAT3 levels upon BMX inhibition (Supplemental Figure 2L). Altogether, these results emphasize the role of *BMX* as a determinant of the viability and self-renewal potential of NB spheroids and provide a rationale for its pharmacological targeting in metastatic nMNA NB tumors.

### BMX inhibition reverts the MES phenotype of NB tumors and increases their sensitivity to chemotherapeutic agents.

**Figure 6:**
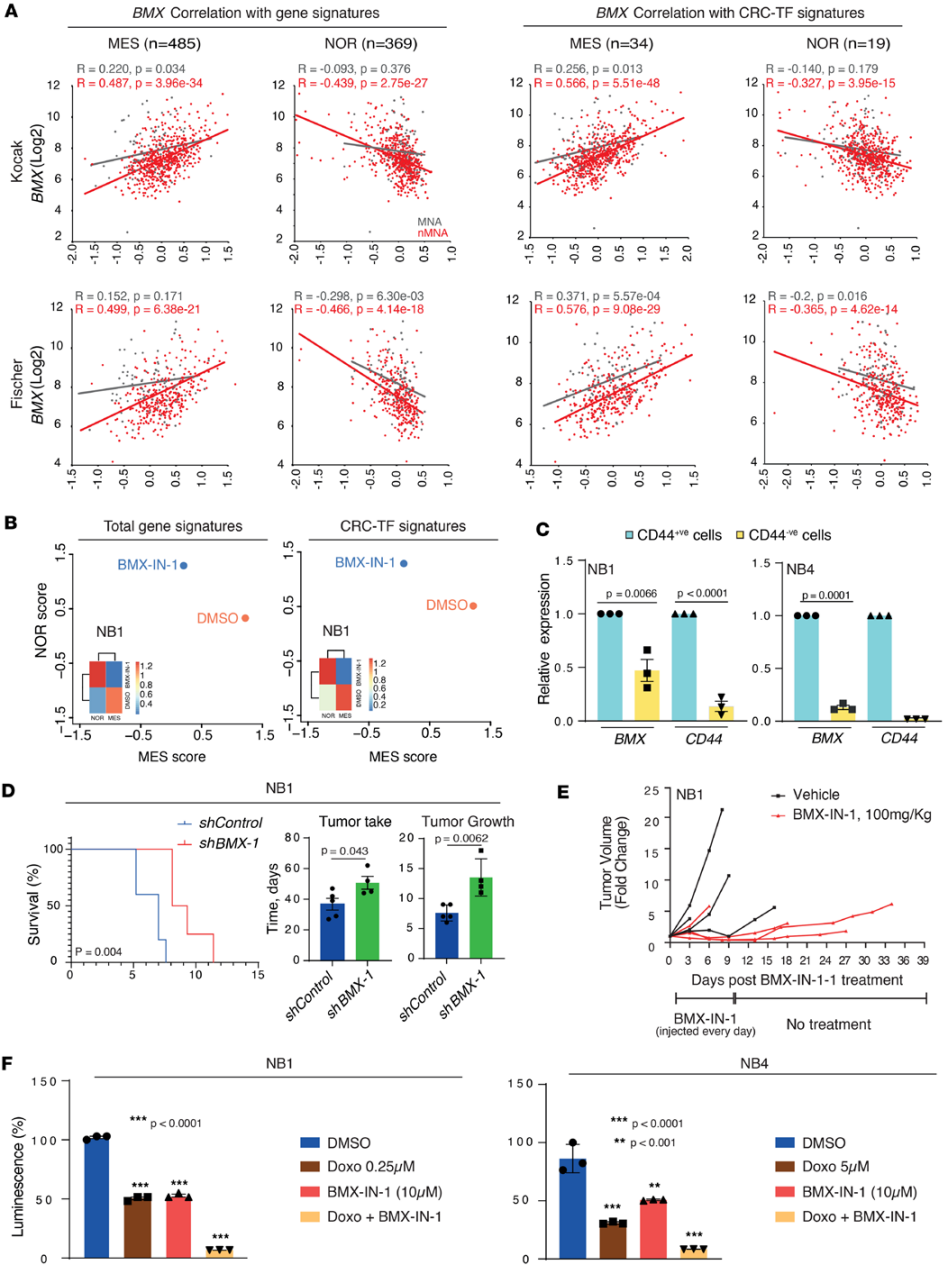
BMX depletion and pharmacological inhibition revert the MES phenotype and decrease the tumorigenic potential of NB spheroids.(A) Scattered plots showing the correlation between BMX expression and the total MES (*n* = 485) or NOR (*n* = 369) gene signature (left panels) or the CRC-TFs of the MES (*n* = 34) or NOR (*n* = 19) cellular states (right panels) in primary tumors from the Kocak and Fischer cohorts (R2 genomics platform). (**B**) Scattered plot and heatmap showing the changes in cell phenotype upon 3 days of BMX-IN-1 (10 μM) treatment for NB1 spheroids versus DMSO. Scores calculated using the total (left panel) or the restricted CRC-TFs (right panel) MES and NOR gene signatures used in **A**. (**C**) Bar graphs illustrating BMX and CD44 mRNA expression levels in CD44^+^ and CD44^–^ NB1 (left) and NB4 (right) populations sorted by FACS. Relative mean expression ± SEM values of 3 technical replicates are shown (unpaired *t* test). (**D**) Kaplan-Meier curves showing improved survival for mice injected with *BMX*-depleted NB1 spheroids (sh*BMX#1*; *n* = 4), relative to *BMX*-proficient NB1 spheroids (shControl; *n* = 5) (left panel, Log-rank test). Bar graphs depicting the time required for tumor establishment (middle panel, number of days to reach approximately 500 mm^3^) and tumor growth (right panel, number of days required to reach the maximal volume from approximately 500 mm^3^) (unpaired *t* test). (**E**) Graph showing the changes in tumor volume for each individual mouse with NB1-derived xenografts treated daily i.p. with either BMX-IN-1 (100 mg/kg, *n* = 4, red lines) or an identical volume of vehicle (*n* = 4, black lines) for 10 days (D0 to D9), and then allowed to grow without treatment until reaching the maximal volume authorized. (**F**) Bar graph showing changes in spheroids viability upon treatment with Doxorubicin alone, BMX-IN-1 alone, or both agents in combination for 1 day or 3 days for the NB1 or NB4 model, respectively. Mean ± SEM values of 3 technical experiments are shown. One-way ANOVA followed by post hoc pairwise comparisons was performed to compute adjusted *P* values. **P < 0.001, ***P < 0.0001.

Given the current need for cell state–specific targeted therapies in NB tumors, we explored the possibility that *BMX* expression, in addition to support the undifferentiated state of NB tumor cells, may also be involved in the establishment/maintenance of these divergent tumor cell identities. To this purpose, we used previously established MES (*n* = 485 genes) and NOR (*n* = 369) gene signatures that define each cell phenotype (19) to survey 4 primary NB transcriptomic data sets for correlation with *BMX* expression levels. Interestingly, we found that *BMX* expression correlates with the MES signature in the 4 cohorts analyzed and anticorrelates with the NOR signature in 3 of 4 patient data sets (Figure 6A and Supplemental Figure 3A). These findings held true when we restricted the gene signatures to CRC-TFs responsible for maintaining the cell identity of MES and NOR cells in NB (Figure 6A and Supplemental Figure 3A) (43).This trend was particularly evident in nMNA tumors, as compared with MNA tumors, consistent with a more prevalent role for *BMX* in shaping cellular phenotypes in the absence of *MYCN* amplification. More importantly, when the gene expression profiles of NB1 and NB4 spheroids treated with BMX-IN-1 were interrogated for changes in the MES or NOR signatures, we found that BMX inhibition efficiently decreased the MES phenotype in both models and increased the NOR state in NB1 spheroids (Figure 6B and Supplemental Figure 3B). Importantly, these trends persisted when analyzing the signatures restricted to CRC-TFs gene signatures (Figure 6B and Supplemental Figure 3B).

Recently, PRRX1 was identified as a master CRC-TF regulating the NOR-to-MES transition in NB cells, and its expression is reported to induce a MES phenotype (19). We therefore interrogated *BMX* expression levels in the NOR SK-N-Be2c NB line and its PRRX1-overexpressing counterpart, which show a transition toward a MES phenotype (19). *BMX* transcript levels strongly increased upon PRRX1 expression in this model (Supplemental Figure 3C), confirming a link between tumor cell phenotypes and *BMX* expression in NB. These differences were further reflected into distinct sensitivities toward BMX-IN-1 between SK-N-Be2c and SK-N-AS adherent cultures, which represent NOR and MES NB phenotypes, respectively (Supplemental Figure 3, D, E and F).

Finally, we prioritized the genes associated with either the NOR or the MES signatures (19) for their correlation score with *BMX* expression in our tumors, spheroids, and adherent NB models using our RNA-Seq data sets (Figure 2A). Through this analysis, we identified *CD44* as the second-most positively correlated gene to *BMX* expression (*R* = 0.89). Given that CD44 has recently been proposed as a prototypical marker defining the MES cell state in NB (43), we dissociated and sorted NB1 and NB4 spheroids by FACS into CD44^+^ and CD44^–^ populations (Supplemental Figure 3G) and assessed their respective *BMX* expression levels by quantitative PCR (qPCR). This approach revealed that, in both tumor models, CD44^+^ NB cells exhibited markedly higher *BMX* expression levels as compared with their CD44^–^ counterparts (Figure 6C).

Collectively, these observations point to a potential role of BMX in supporting the NB MES phenotype and suggest that BMX inhibition could represent an additional approach to improve current therapeutic strategies, particularly in chemoresistant nMNA tumors.

### BMX depletion decreases the tumor initiation properties of NB spheroids.

Our in vitro results so far indicate that BMX play a major role in maintaining the self-renewal ability, viability, and MES phenotype of NB spheroids. Based on these observations, we decided to assess the effect of depleting *BMX* expression on the tumor-initiation properties of these models. For in vivo experiments, we selected the NB1 model, given its higher *BMX* expression levels and more pronounced MES to NOR phenotype transition upon BMX inhibition, and injected 2 million spheroid cells transduced with either control or *BMX*-targeting shRNA sequences (sh*BMX*#1) s.c. into the flank of 4- to 8-week-old NOD/SCID/γ c (NSG) mice. Mice were sacrificed when tumor size reached the maximal tumor volume authorized. Consistent with our in vitro findings, *BMX* depletion resulted in prolonged survival for mice injected with NB1 spheroid cells, reflecting a marked delay in tumor establishment and growth (Figure 6D). To further support these findings, we next assessed the preclinical relevance of BMX-IN-1 in vivo. NSG mice were injected s.c. with 2 million cells derived from NB1 spheroids, and tumors were allowed to grow until reaching a 500 mm^3^ volume. At this point, mice were treated every day with either 100 mg/kg of BMX-IN-1 or an equivalent volume of vehicle over a period of 10 days (D0 to D9), after which tumors were allowed to grow untreated until they reached the maximal tumor volume authorized. Notably, 3 of the 4 mice of the Control group had reached the maximal volume before receiving all planed treatments (sacrificed at D3, D8, and D9), whereas only 1 mouse of the BMX-IN-1 group did so (D6) (Figure 6E). Moreover, after the end of treatment, BMX-IN-1–treated tumors still displayed a tendency to grow more slowly than the remaining tumor of the Control group. Thus, consistent with the in vivo results obtained using shRNA lentiviral vectors, pharmacological inhibition of BMX also results in reduced NB1 tumor growth and prolonged mice survival (Figure 6E).

Despite the decrease in tumor growth observed with BMX-IN-1 as a single agent, we reasoned that the MES-to-NOR phenotypic transition induced by BMX inhibition may constitute an opportunity for synergistic therapeutic combinations, given that the resistance of NB tumors toward conventional chemotherapy is often attributed to their enrichment in MES cells. We therefore considered that combining BMX inhibition with chemotherapeutic agents may represent a clinically relevant strategy, by increasing NB sensitivity toward the current standard-of-care regimens. To validate this hypothesis, we investigated whether BMX inhibition could increase the sensitivity of NB1 and NB4 spheroids toward the standard-of-care chemotherapeutic agent Doxorubicin. Initially, a time-dependent analysis of NB1 and NB4 spheroid cell sensitivity toward Doxorubicin was performed. NB1 spheroids demonstrated pronounced sensitivity to Doxorubicin (0.25 μM, < 50% of viable cells at D1), as compared with NB4 spheroids (5 μM, < 50% of viable cells at D3; Supplemental Figure 3H), consistent with the origin of the NB4 model from a patient who experienced disease relapse after chemotherapy. Subsequently, both spheroid models were treated with either BMX-IN-1 or Doxorubicin alone or with a combination of the 2 agents. Interestingly, the combination of BMX-IN-1 and Doxorubicin yielded a strong therapeutic effect, with less than 10% of viable spheroid cells remaining compared with single-agent treatment (50%). This effect was particularly notable in NB4 spheroids, which are known for their resistance to Doxorubicin, reflecting the chemorefractory state seen in patients with NB (Figure 6F).

Taken together, these findings underscore the crucial role of BMX in promoting the MES and chemoresistant cellular phenotype in metastatic nMNA NB tumors. Therefore, targeted BMX inhibition, in combination with chemotherapy, presents a viable strategy to enhance the response of patients with metastatic NB to the current standard-of-care regimen.

## Discussion

The past decade has witnessed the rapid evolution of next-generation precision medicine, which hold promise to provide new and patient-centered therapies. Most of the current efforts in this field, however, focused on genome-guided therapies and have provided substantial benefits to only a limited subset of patients bearing actionable mutations. Given our increasing understanding of the importance of additional drivers of malignancy during tumor progression and response to therapy, it has become evident that future efforts in this field should include multilayered analyses of the tumor ecosystem, including epigenetic alterations affecting tumor cells and their microenvironment. This approach requires complex cellular models that faithfully recapitulate the main features of patient’s tumors and can be interrogated to scale in order to deliver new treatments for patients with refractory or advanced diseases. Among them, 3D patient-derived tumor models have recently emerged as a model of choice to interrogate tumor behaviors while maintaining most of the key biological features of their tumor of origin (44). In particular, 3D patient-derived models have shown great potential in unravelling key determinants of tumor cell plasticity in vitro, with direct functional implication to deciphering new mechanisms involved in establishing and maintaining tumor heterogeneity.

Plasticity promotes tumor initiation and dissemination, while permitting tumor cells to transition to resistant cell states by epigenetic reprogramming, and it is also a requisite for the emergence of intratumor heterogeneity (45–47). Because of the direct link between tumor heterogeneity and the acquisition of intrinsic therapeutic resistance, targeting cell plasticity and/or specific tumor cell states is an increasingly and clinically relevant field of investigation (48, 49). Importantly, both genetic and epigenetic alterations participate in the establishment of the tumor phenotype, with changes in chromatin states supporting rapid alterations in transcriptional programs and related oncogenic competences that follow tumor progression and response to therapy (50). These notions highlight the importance of selecting the most appropriate model to interrogate tumor heterogeneity and define potential therapeutic interventions to blunt cellular reprogramming and plasticity in cancer. They are also particularly relevant in pediatric malignancies, where the intrinsic heterogeneous nature of the disease, paired with a relative lack of druggable genetic mutations, requires the ability to identify and target the most aggressive subpopulations of tumor cells.

NB is a heterogeneous disease with a median survival of less than 50% for HR-NB even after multimodal therapy, testifying for the little improvement experienced by these patients over the last decades. Among the most important reasons for this are increased tumor heterogeneity, cellular plasticity, and related resistance to therapy observed in these diseases. Consistent with this, HR-NB tumors are enriched for cell subpopulations endowed with stem-like features, including tumor-initiating and propagating abilities, and which are responsible for tumor relapse and resistance to therapy (51). Optimal strategies for these patients should therefore include targeting multiple coexistent cell states/phenotypes within the same tumor, and the critical pathways regulating their plasticity. In keeping with this, functional screens of 3D NB models from metastatic stages may yield important biological insight on mechanisms governing intratumoral heterogeneity and response to therapy in NB.

Following this strategy, in our study, we show for the first time to our knowledge that the nonreceptor tyrosine kinase (TK) BMX is preferentially expressed in undifferentiated NB metastatic cells and regulates their clonogenicity, MES phenotype, and tumor-initiating potential. Interestingly, single nuclei transcriptomic analyses of human postnatal adrenal glands and primary NB tumors identified *BMX* expression in the progenitor hC1 adrenal cluster and the undifferentiated nC3 malignant cluster characteristic of HR-NB, respectively (52). Both clusters shared similar gene signatures with high expression of mesenchymal, migratory, and progenitor-related genes. Consistent with our observations on the role of BMX in maintaining the MES and undifferentiated phenotype in primary NB models, the malignant nC3 cluster was shown to express PRRX1 and other markers of the MES signature (19, 20). *BMX* is a nonreceptor tyrosine kinase member of the Tec family and contains several essential functional domains, including a Pleckstrin homology (PH) and Tec homology (TH) domain, as well as multiple Src homology domains (SH1–3) (53). *BMX* expression is elevated in multiple tumor types, including prostate (54), cervical (55), renal (56), and colorectal carcinoma (57), as well as glioblastoma (58). From a functional standpoint, BMX was shown to modulate chemoresistance of small cell lung cancer cells (SCLC) (59) and promote proliferation of prostate cancer cell lines (38). Importantly, BMX expression was also reported to be essential for the tumor-initiating ability of glioblastoma stem cells (37). Taken together, these observations identify BMX as an attractive therapeutic candidate for different tumor types, with potential roles in tumor growth, differentiation, and response to therapy.

Molecular profiles and clinical stages of NB have been well defined, but the identification of druggable targets in addition to ALK is challenging due to the lack of recurrent mutations and to the intratumor biological variations (7–9). *MYCN* amplification is one of the major critical molecular features to stratify HR-NB, and its role in NB development and progression is established (60). However, fewer studies focused on determining the factors contributing to the pathogenesis of HR nMNA NB, limiting the development of novel treatment strategies against these tumors. Given that deregulated *MYCN* expression is known to dominate the active *cis*-regulatory landscape of MNA NB tumors (34), we therefore reasoned that focusing on nMNA tumors may provide us with a better opportunity to identify additional oncogenic factors promoting the most aggressive state of this disease. In line with this, an intriguing observation in our study is the differential sensitivity between MNA and nMNA NB tumors toward *BMX* depletion, despite similar expression levels. These results suggest that this kinase represents a selective vulnerability for tumors lacking *MYCN* amplifications, whereas in MNA tumors, this pathway may be overridden by the powerful activity of *MYCN* and its direct targets, which may compensate the deleterious effects of *BMX* depletion. An alternative, and nonmutually exclusive, scenario could indicate that transcriptional programs sustaining the undifferentiated populations in NB are distinct between MNA and nMNA tumors, with BMX playing as preeminent role in the latter category. These observations raise important questions about the design of cell state–specific therapies in this setting and suggest that a more holistic analysis of NB tumors that includes cell phenotypes, genotypes, and epigenetic states would improve the design of next-generation therapeutic strategies.

From a translational perspective, it is tempting to speculate that BMX inhibition could synergize with current standard of care and targeted therapies to eradicate the most aggressive tumor subpopulations in advanced and refractory NB. Although our data seem to support this possibility for the chemotherapeutic agent Doxorubucin, additional studies are warranted to better define the potential synergistic effect of BMX inhibitors and the functional underpinnings of their activity. Multiple nonmutually exclusive mechanisms may explain these results. First, BMX inhibitors have the ability to reverse the MES phenotype, which has been associated with increased resistance to multiple therapies (19) and may increase tumor sensitivity to selected agents. Second, the induction of antiapoptotic signals by BMX are observed in different cancer types, including *BCL2*/*BCL-xl* expression in SCLC (61), and PIM-1 expression in prostate cancer (62). It would be interesting to investigate if similar mechanisms are also actively involved in NB. Finally, it would be interesting to investigate if the effect we observed combining BMX inhibitor and Doxorubicin also applies to other conventional chemotherapeutic agents used for NB treatment as well as to targeted therapies like ALK inhibition, where the MES phenotype has been shown to play major roles in tumor resistance (22).

A potential limitation of the current study is the lack of TME components in our in vitro models, preventing us from further characterizing the effect of BMX inhibition on stromal and immune cells. This may be particularly relevant in light of *BMX* expression by endothelial cells (63) and the role played by this kinase in enhancing tumor angiogenesis through the PI3K/Akt angiogenic signaling pathway, independent of VEGF (64). However, our in vivo results using both shRNA and pharmacological agents tend to confirm that *BMX* depletion/inhibition within a complex tumor ecosystem still leads to substantial decrease in tumor growth, supporting the translational relevance of our findings. It would also be interesting to spatially resolve the distribution of BMX-expressing cells in primary human NB tumors across different stages and to assess if BMX is preferentially expressed in any defined location within the tumor, similar to the perivascular BMX^+^ niches observed in GBM (37). A second limitation is the lack of high-resolution data able to provide a detailed understanding of cell state transitions induced by *BMX* depletion. Future single-cell RNA-Seq (scRNA-Seq) profiling of NB spheroids treated with BMX inhibitors will help delineate how different cell types within the same spheroid may transition to a NOR state or decrease their proliferation and viability. Finally, our relatively limited in vivo data currently precludes a definitive assessment of the clinical translatability of our findings and warrants additional preclinical modeling to better define the potential value of BMX inhibition in NB.

Altogether, our observations indicate that BMX inhibition could offer a relevant antitumor strategy for patients with HR-NB, with limited on-target toxicity given the modest phenotype displayed by murine KO models (65), and may be particularly efficient in combination with other targeted therapies aiming at eradicating the distinct cell states that participate in the emergence of therapeutic resistance and compose the ecosystem of metastatic NB tumors.

## Methods

### Sex as a biological variable.

Sex was not considered as a biological variable. Both primary NB1 and NB4 models and the SK-N-Be2c cell line were derived from male patients while the SK-N-AS cell line originates from a female patient. For in vivo studies, only female mice were used based on practical considerations.

### Isolation of NB primary cells and establishment of spheroids from patient samples.

The NB1 and NB4 PDX models derived from metastatic cells aspirated from the bone marrow of patients with stage 4 HR-NB at diagnosis (NB1) or at relapse (NB4) have been previously published (31). NB-PDXs were maintained in vivo by serial s.c. implantations of either small tumor fragments or dissociated xenograft cells into the flanks of 4- to 8-week-old female athymic Swiss nude mice (Charles River Laboratories) or NSG mice (The Jackson Laboratory). For implantations of primary NB cells, 0.8 × 10^6^ to 2 × 10^6^ cells were suspended in 200 μL of DMEM (Invitrogen) and BD Matrigel Basement Membrane matrix (1:1; BD Biosciences). Tumor growth was followed up using calipers every 3 days. Mice were sacrificed once tumors reached a volume of approximately 900 mm^3^ and a single-cell suspension was prepared as follow before switching to spheroids or adherent cell culturing conditions. The NB-PDX samples were subjected to mechanical and enzymatic dissociation using Tumor Dissociation Kit (Miltenyi Biotec). For in vitro experiments, tumor tissue was engrafted into the flanks of NSG mice, and the animals were sacrificed once tumor reached the volume of 1 cm^3^. Tumors were subjected to mechanical and enzymatic dissociation using the Tumor Dissociation Kit (Miltenyi Biotec). For spheroid culture, the dissociated tumor cells were suspended in Neuro basal medium (NBM): DMEM/F12 medium supplemented with 1% penicillin-streptomycin, 2% B27 (Invitrogen), and 20 ng/mL of human recombinant FGF and EGF (Peprotech) and grown in ultralow attachment plates (Corning), Adherent cells were generated from matched spheroids using DMEM containing 10% FBS and supplemented with 1% penicillin-streptomycin. Human embryonic kidney (HEK) 293T cells (ATCC) and the adherent cells were cultured in DMEM (Thermo Fisher Scientific) supplemented with 10% of FBS (PAN-Biotech) and 1% of penicillin-streptomycin (Thermo Fisher Scientific). Cells were maintained at 37°C and 5% CO_2_ in humidified chambers.

### RNA-Seq.

For tumor samples, dissociation was performed following gentleMACS Dissociator kit instructions for soft tumors (Miltenyi Biotech). Total RNA was extracted with miRCURY kit (Exiqon) using Lysis buffer + B-mercaptoethanol. Minimum 100 ng used for preparing sequencing libraries using the TruSeq mRNA stranded kit (Illumina) and sequencing was performed on a Hi-Seq Illumina Genome Analyzer (100 bp single-read).

RNA-Seq reads were mapped to the hg38 genome (only chromosomes 1–22, X, Y and M) using hisat2 (version 2.0.5) and the Ensembl gene annotations Homo sapiens GRCh38.87. Read density tracks were generated using bedtools genomecov (version 2.29.0 option -split) for each genomic position, and read counts were normalized to 1 million reads in the library and visualized using the IGV browser. Raw read counts and RPKM values were calculated over gene exons using htseq-count (version 0.9.1). Differentially expressed genes were identified using DESeq2 (version 1.26.0) using the following thresholds: adjusted *P* ≤ 0.001 and fold change ≥ 2. Gene ontology annotations were used to calculate the enrichment of biological processes and associated hypergeometric *P* values of genes in each class compared with all genes.

### ChIP-Seq.

Single cells from adherent or spheroids cultures were counted and diluted to a target of 500,000 cells per mL. Formaldehyde at 1% final concentration was added for 10 minutes at 37°C. Quenching was performed with 125 mM Glycine. The solution was spun at 225*g* for 5 minutes at 4°C, and the pellet washed twice with cold PBS + protease inhibitors (PI) before snap freezing. For tumors ~40 mg, a fragment was put in petri dish on normal ice, cut into small pieces and resuspended in 1 mL PBS + protease inhibitors, and transferred to a 1.5 mL tube. Formaldehyde at 1% final concentration was added for 10 minutes at 37°C. Quenching was performed with 125 mM Glycine. The pellet was washed once in cold PBS + PI after a 5-minute spin at 625*g* at 4°C. Manual dissociation in PBS + PI was performed with syringe passages: 10 up/down with a 18G needle, 10 up/down with a 21G needle, and 10 up/down with a 23G needle (if possible).

The solution was spun down another time, and the pellet was snap frozen. Lysis was performed at 4°C for 10 minutes in 300 μL of 50 mM Tris-HCl, 1% SDS, and 0.25% DOC (+ PI), and sonication was performed with tip sonicator after dilution in 900 μL of dilution buffer (DB) — 50mM Tris-HCl (pH 7.4), 0.1% SDS, 150 mM NaCl, 1.84% Triton-X + PI — with the following parameters: sonication time, 3 minutes; amplitude, 40%; cycle, 0.7 seconds on and 1.3 seconds off. After clearing the solution with 19,600*g* spin, the supernatant was further diluted with 1.8 mL of DB before i.p. overnight at 4°C. The equivalent of 2 million to 5 million cells or 5–10 mg of tumors was used for a single i.p. with 0.1–1 μg H3K4me3 antibody (MilliporeSigma, 07–473).

After washing once Protein G magnetic beads in DB, the equivalent of 30 μL (50 μL for tumors) of original solution was added to the IPs for 2 hours at 4°C. Beads were washed 2× with 150 mM NaCl RIPA buffer, 500 mM NaCl RIPA, LiCl buffer (10 mM Tris-HCl [pH 8.1], 250 mM LiCl, 0.5% Triton X-100, 0.5% DOC) and once in 10 mM Tris-Cl (pH 8.5). Elution was performed with 1× TE (pH 8.0), 0.1% SDS, 150 mM NaCl, 5 mM DTT at 65°C for 1 hour. RNase treatment and crosslink reversal with proteinase K were applied on the supernatant, before SPRI bead DNA purification. After Qubit quantification, 5–10 ng DNA was used for Illumina TruSeq Library preparation. Sequencing was performed on a Hi-Seq Illumina Genome Analyzer (50 bp single-read).

ChIP-Seq reads were trimmed using trim_galore (version 0.6.4 options -q 20 –stringency 2), mapped to the hg38 genome (only chromosomes 1–22, X, Y, and M) using bowtie2 (version 2.3.0), and only reads with mapping quality ≥ 10 were kept. Read density tracks were generated using bedtools genomecov (version 2.29.0) for each genomic position from reads extended to 200 bp, and read counts were normalized to 1 million reads in the library and visualized using the IGV browser. Peaks were called using MACS2 (version 2.2.5 option --broad-cutoff 0.00001), and peaks located on chromosome M or in ENCODE blacklisted regions were removed. For comparison across samples, ChIP-Seq peak regions were merged using bedtools merge (version 2.29.0), and raw read counts per region were calculated using bedtools intersect (version 2.29.0 option -c). Differentially enriched regions were identified using DESeq2 (version 1.26.0) using the following thresholds: adjusted *P* ≤ 0.05 and fold change ≥ 2.

### Lentivirus production and transduction.

HEK 293T cells (ATCC) were cultured, as mentioned above. Approximately 10 million cells were seeded in a 150 mm cell culture dish (Thermo Fisher scientific); cell conditions were checked and transfected the following day using FuGene6 (Promega). pMD2G (Addgene, 12259) and pCMVΔR8.74 (Addgene, 12263) vectors were used as envelope and packaging plasmids, respectively. After 48 hours, viral supernatant was collected and filtered using a Millipore filtration system (0.45 μm). The filtered supernatant was concentrated using Lenti-X Concentrator (Clontech-Takara), and the virus-containing pellets were resuspended using serum-free media and added on cells (single cells were obtained from spheroids after treating with Accutase) in the presence of media containing 6 μg/mL polybrene. Depletion of *BMX* was achieved by using pLKO.1 lentiviral shRNAs purchased from the RNAi Consortium (sh*BMX*#1 [TRCN0000006359]; sh*BMX*#2 [NB1, TRCN0000006363]; sh*BMX*#2 [NB4, TRCN0000006362]). Control cells were infected with shRNA targeting the GFP transcript (5′ - GCAAGCTGACCCT-GAAGTTCAT - 3′).

### RNA extraction and real-time PCR.

Total RNA was extracted using the RNeasy Mini Kit (QIAGEN) as per the manufacturer’s protocol and recommendations. Five hundred nanograms of total RNA were used for cDNA conversion using the high-capacity cDNA conversion kit (Applied Biosystems). Real-time PCR was performed using PowerUp SYBR Green PCR Master Mix (Invitrogen) in a QuantStudio 5 System instrument (Thermo Fisher Scientific) using the following primers: *BMX*-forward 5′ - TGA CTG GTG GCA AGT AAG AAA ACT - 3′ and reverse 5′ - ACC AGC AAA CCAGTC ATA ATC ATC C - 3′; *GAPDH*-forward 5′ - AGC CAC ATC GCT CAG ACA C - 3′ and reverse 5′ - GCC CAA TAC GACCAA ATC C - 3′. The PCR cycle conditions included an initial holding period at 50°C for 2 minutes and 95°C for 10 minutes, followed by 95°C for 15 seconds and 60°C for 1 minute for 40 cycles. Relative quantitation of gene expression data was conducted according to the 2^−ΔΔCt^ method. GAPDH was used as reference control gene. Statistical analysis was performed by unpaired, 2-tailed Student’s *t* test.

### Cell viability assay.

NB spheroids were subjected to accutase treatment to obtain a single-cell suspension. After 8 hours of viral transduction (shControl and sh*BMX*), 6,000 cells/well were seeded in an ultralow attachment 96-well plate in NBM medium supplemented with 1% penicillin-streptomycin, 2% B27 (Invitrogen), and 20ng/mL of human recombinant FGF and EGF (Peprotech). After 4 days, cell viability was analyzed using the CellTiter-Glo Luminescent Cell Viability Assay kit (G7571, Promega Corporation) according to the manufacturer’s protocol. This luminescence-based assay quantifies cellular metabolic activity through ATP levels. Endpoint luminescence was measured on a SpectraMax M5 plate reader (Molecular Devices). The protocol was the same for the time-course experiment. The cells were seeded in media containing DMSO and BMX-IN-1 (MedChem Express) and subjected to analysis, as described previously. Statistical analysis was performed by Student’s *t* test.

### RNA in situ hybridization (ISH).

The RNAscope 2.5 HD Duplex Assay (Bio-techne, 322500) was performed according to manufacturer’s protocol on 4 μm paraffin sections, hybridized with the probe Hs-BMX-No-XMm-C1 (Bio-techne, 1275131) at 40°C for 2 hours. The signal from the C1 channel, indicating the presence of the target RNA, was visualized using green fluorescence. Tissues were counterstained with Mayer hematoxyline for 10 seconds, dried at 60°C for 15 minutes, cleared, and mounted with Vectamount (Vector labs, H-5000).

### Sphere-formation assay.

NB spheroids were first subjected to Lentivirus infection to knock down the expression of *BMX*. Once the knockdown was confirmed, the cell suspension from the Spheroid cultures was subjected to a single cell (live) sorting into 3 ultralow attachment 96-well plates (Corning) for each experimental condition using a Moflo Astros EQ cell sorter (Beckman Coulter). Calcein AM (Thermo Fisher Scientific) was used for live-cell detection. Sphere formation was monitored and scored 6 weeks later. For the drug experiments, which involved testing the effects of BMX inhibitor BMX-IN-1 on spheroid growth, single live cells were sorted into ultralow attachment 96-well plates containing either DMSO or 10μM of BMX-IN-1. The spheroids were maintained in either DMSO or BMX-IN-1influence until the end of the experiment.

### Image-based assessment of NB spheroid morphology and viability upon BMX inhibition.

Spheroids were manually dissociated into single cells using Accutase. Around 10,000 cells/well were seeded in a 96-well ultra-low attachment plate format (Corning). The cells were maintained in the incubator for a week in the presence of DMSO (negative control), Gambogic acid 1 μM (positive control), and BMX-IN-1, 10 μM. On the day of analysis, the cells were labeled with Calcein AM and Ethidium homodimer-1 according to the manufacturer’s protocol (Live/DEAD Viability/Cytotoxicity Kit for mammalian cells, L3224, Thermo Fisher Scientific) for 1 hour, followed by additional staining with Hoechst 33342 (Sigma-Aldrich), and were live imaged using an INCell Analyzer 2200 (GE Healthcare). The automated acquisition was performed in an environmental chamber (at 37°C with 5% CO_2_) using a 4×/0.2 NA objective, allowing imaging of the entire well surface by acquiring 4 field-of-view, while still allowing precise segmentation of individual cells, nuclei, or regions of interest.

All the images were analyzed using either pipelines in CellProfiler v4.2.1 or custom-made macros in Fiji, and extracted data were processed either through the EPFL-BSF in-house LIMS or through workflows in Knime v4.5.1 (https://www.knime.com/, accessed on 20 January 2022). Image analysis consisted of, first, an illumination correction to compensate for uneven illumination/lighting/shading, followed by rescaling of image intensities as preprocessing steps. Then spheres/individual cells were segmented using 3-class Otsu’s thresholding of an intermediate image composed of both Calcein AM and Ethidium homodimer-1 signals (to avoid missing objects positive in only 1 of the channels). Finally, many features were extracted and quantified from the segmented objects, with the assessment of size, shape, intensity, and granularity information of all segmented objects. Each plate contained replicates of positive and negative controls (16 replicates for each condition), and all results were normalized according to the DMSO control of the corresponding plate.

### In vivo experiments, cell sorting, and tumor monitoring.

Animal experiments were approved by the Animal Experimentation Ethics Committee of the Veterinary Service of the Canton of Vaud (Etat de Vaud, Service Vétérinaire, authorization nos. VD2995 and VD3437). The NB spheroids were transduced with Lentivirus to knock down the expression of *BMX*. Three days later, the knockdown was confirmed by real-time PCR analysis. The next day, the cells were treated with accutase to obtain a single-cell suspension. Two million cells derived from spheroid culture were suspended in 100 μL of PBS and injected s.c. to both the flanks of 4- to 8-week-old female NSG mice (The Jackson Laboratory). For the in vivo drug experiments, 2 million NB1 spheroid cells were suspended in 100 μL of PBS and implanted s.c. into the dorsal flank of 4- to 8-week-old female NSG mice (Jackson Laboratory). The mice were injected i.p. with BMX-IN-1 (100 mg/Kg) or vehicle (100% N-methyl-2-pyrrolidone diluted 1:4 with sterile 50% PEG400) at indicated doses once per day. Tumor volume was calculated using the formula: Volume (mm^3^) = (l × w^2^)/2, where w (width) is the smaller and l (length) is the larger of 2 perpendicular tumor axes. The animals were euthanized when the tumor volume reached 2,000 mm^3^. The tumor grafts were fixed in 4% paraformaldehyde and processed for H&E staining.

### Flow cytometry analysis and sorting.

NB1 and NB4 spheroids were initially dissociated into single-cell suspensions using accutase (Thermo Fisher Scientific) and subsequently washed with phosphate-buffered saline. Cells were incubated with a mouse anti-CD44 antibody (clone F.10.44.2; ref. 66) 1:2 dilution in FACS buffer (PBS-1% BSA-2 mM EDTA) for 15 minutes at 4°C. After washes, cells were incubated with fluorescent secondary antibody (goat anti–mouse Alexa 647, Thermo Fisher Scientific, A21236, dilution 1/500 in FACS buffer) for 30 minutes at 4°C, and washed 3× with FACS buffer. DAPI (1:1,000 dilution of 100 μg/mL, Biotium) was added just before sorting to distinguish live from dead cells. The sorting of CD44^+^ and CD44^–^ cell populations was performed using the FACS Aria III cell sorter (BD Biosciences)

### Immunobloting.

NB1 and NB4 spheroid cells were suspended in 2× sample buffer containing Tris-HCl (pH 6.8, 125 mM), SDS (4%), glycerol (20%), bromophenol blue (0.02%), and β-mercaptoethanol (0.1M) previous to sonication. Equal amounts of total protein lysate were loaded onto a 4%–15% precast polyacrylamide Mini-PROTEAN TGX gel (Bio-Rad). Proteins were transferred to a PVDF membrane (Immuno-Blot, Bio-Rad) and blocked with 3% nonfat dry milk in TBS-T (0.1%). The membrane was incubated overnight at 4°C with primary antibodies against STAT3 (Cell Signaling Technology, 9139, mouse, 1:1,000 dilution) and pSTAT3-Tyr705 (Cell Signaling Technology, 9131, rabbit, 1:500 dilution), followed by 1-hour incubation with the appropriate secondary antibodies (goat anti–mouse HRP, Jackson ImmunoResearch, 115-035-166, 1:10,000 dilution or goat-anti rabbit HRP, Agilent, P0448). Protein visualization was performed using either the WesternBright Sirius Kit or the WesternBright Quantum Kit (Advansta Inc.) and the Fusion FX Spectra multimodal imaging platform (Vilber Lourmat).

### Published data.

Published expression data was obtained from the R2 Genomics Analysis and Visualization Platform (https://r2.amc.nl). Expression data sets from Kocak (GEO GSE45547) (67), Fischer (ArrayExpress E-MTAB-8248) (68), SEQC consortium (GEO GSE49710), Maris (GEO GSE89413) (69) and Cangelosi (70) were used.

Published IC_50_ data were obtained from the Genomics of Drug Sensitivity in Cancer Platform (https://www.cancerrxgene.org/).

### Statistics.

GraphPad Prism (version 7) was used to generate graphs and perform unpaired, 2-tailed *t* test or 1-way ANOVA. Additionally, a 1-way ANOVA followed by post hoc pairwise comparisons to compute adjusted *P* values was performed whenever necessary, ensuring a more stringent assessment of the observed differences across conditions. qPCR data were collected by QuantStudio design and analyses software (version 1.4.2). All genomic analyses were performed using custom scripts in unix using awk and bedtools and R for plots and statistics. Adobe Illustrator (2020) was used to create the figures. A *P* value below 0.05 was deemed significant.

### Study approval.

Patient samples were collected at the Hemato-oncology Unit of the University Hospital of Lausanne (Switzerland) after informed consent and with the approval of the ethical committee of the Canton de Vaud NB1 and NB4 (VD 26/05), Ewing sarcoma and Synovial sarcoma (VD 260/15). All in vivo procedures were performed under the guidelines of the Swiss Animal Protection Ordinance and the Animal Experimentation Ordinance of the Swiss Federal Veterinary Office (FVO). Animal experiments were approved by the Animal Experimentation Ethics Committee of the Veterinary Service of the Canton de Vaud (authorization nos. VD2995 and VD3437). All reasonable efforts were made to reduce suffering, including anesthesia for painful procedures.

### Data availability.

The raw data generated in this study have been deposited in Zenodo (https://doi.org/10.5281/zenodo.7431535). The processed data generated in this study have been deposited at the Gene Expression Omnibus (GEO) under the accession no. GSE223902. Values for all data points in graphs are reported in the Supporting Data Values file.

## Author contributions

SS, DFC, AMM, AFB and NR designed the study and wrote the manuscript. AMM, RR, GT, MNR and IS provided necessary reagents and conceptual advice. SS, DFC, LB, LC, RS, KBB, GB, FK, performed the experiments. SS and AFB conducted bioinformatic analyses.

## Supplementary Material

Supplemental data

Unedited blot and gel images

Supplemental tables 1-3

Supporting data values

## Version 1 07/22/2024

Electronic publication
