# Supplementary material for: Preclinical spheroid models identify BMX as a therapeutic target for metastatic MYCN nonamplified neuroblastoma
Source: JCI Insight. 2024 Jul 22;9(14):e169647. doi: 10.1172/jci.insight.169647 (PMC11383371; doi:10.1172/jci.insight.169647)
Supplement: Supplemental data [file jciinsight-9-169647-s200.pdf]

Supplementary Figure 1

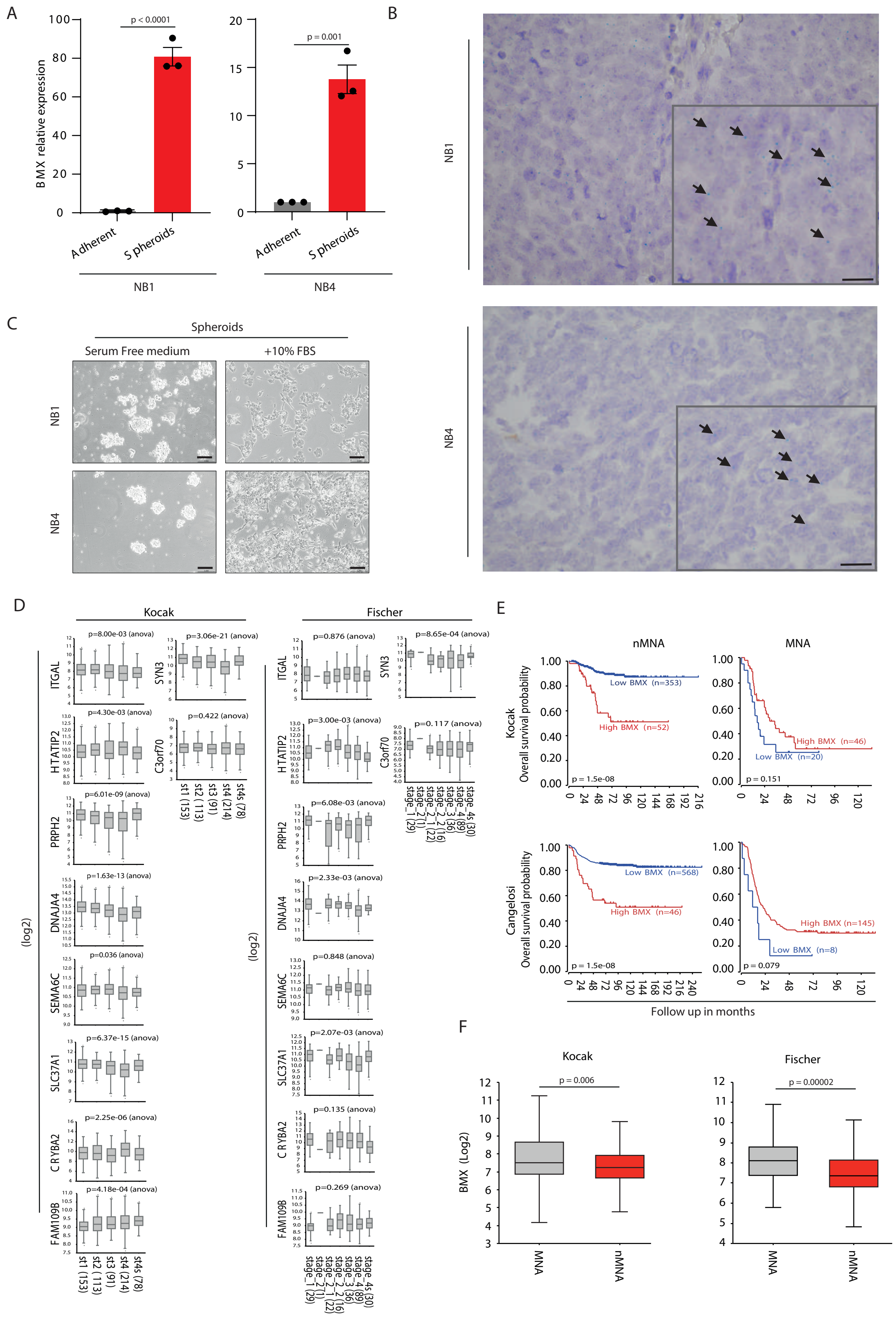

Supplementary Figure 1: BMX expression is elevated in primary NB spheroids and associated with lower survival of nMNA NB patients.

(**A**) Bar plots depict qRT-PCR assessment of *BMX* transcript levels between matched spheroids and adherent cultures for the NB1 (*left*) and NB4 (*right*) tumor models. Mean ± SEM values of three technical replicates are shown. Statistical analysis was done by unpaired t-test. (**B**) Representative images of RNA-ISH analysis showing *BMX* mRNA expression in NB1 and NB4-derived xenografts. *BMX* mRNA transcripts are visualized as blue dots pointed by the arrows, indicating active expression. (Scale bars, 20 µm. Magnification, 60X). **(C)** Micrographs illustrating representative images of NB1 (*top*), NB4 *(bottom)* spheroids exposed to serum-free or 10% FBS-containing media for eight days. (Scale bars, 100 µm. Magnification, 10X)**. (D)** Box plots showing expression levels for 10 genes included in the signature from Fig. **2H** and derived from primary NB samples included in the Kocak and Fischer NB cohorts from the R2 genomics platform. Patient samples were grouped based on INSS stages 1 to 4s, and statistical analyses were performed using the two-sided unpaired t-test. **(E)** Kaplan-Meier overall survival curves for NB patients from the Kocak (*Top*) and Cangelosi (*Bottom*) cohorts harboring stratified by *BMX* expression levels and their *MYCN* amplification status. The data was obtained from the R2 genomics platform. Statistical analyses were performed using the log-rank test. **(F)** Comparison of *BMX* mRNA expression levels for NB samples from the cohorts as in **(E)**, segregated based on *MYCN* amplification status. Welch p-value is shown.

Supplementary Figure 2

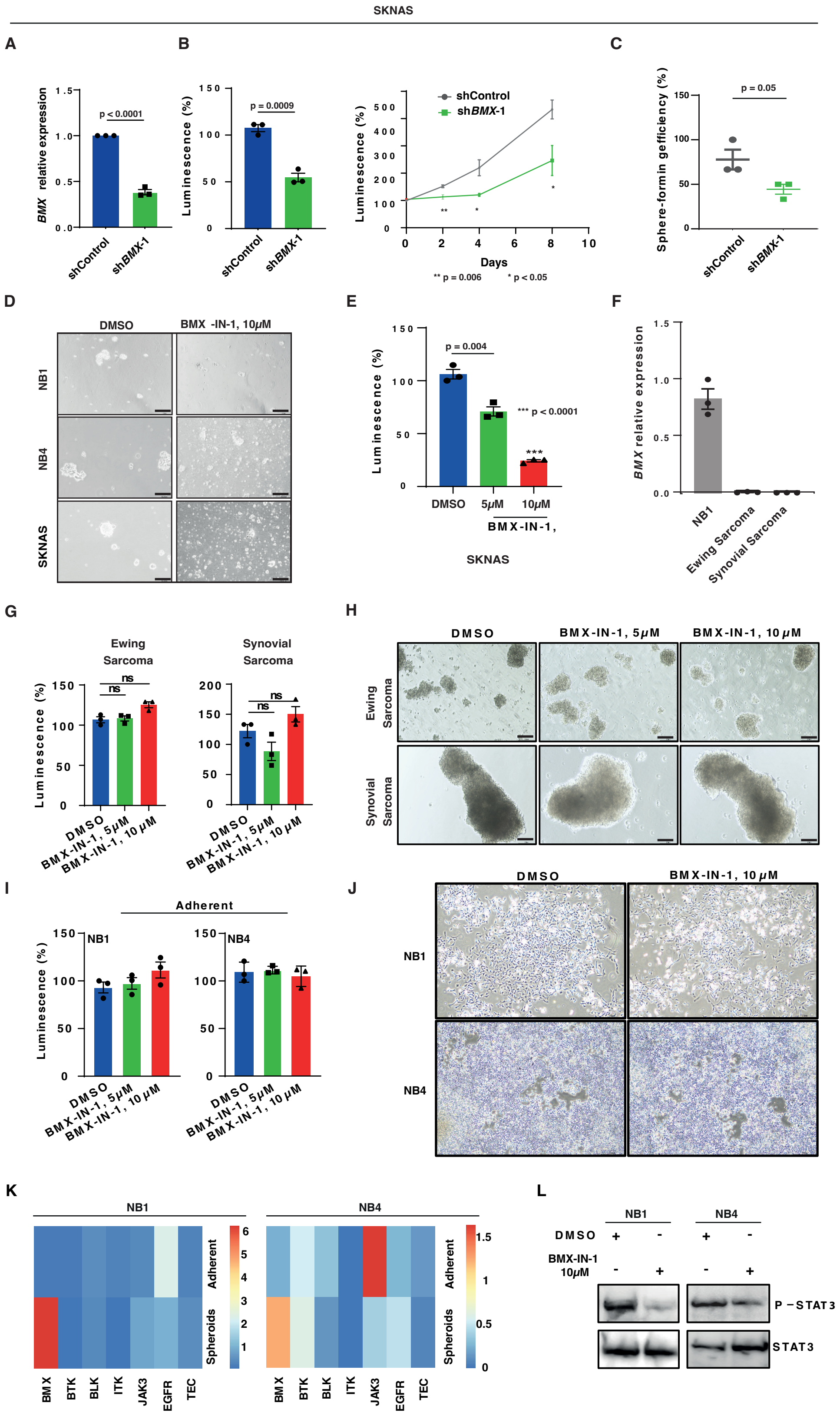

Supplementary Figure 2: The biological effects induced by BMX-IN-1 in NB spheroid models are specifically mediated by on-target BMX inhibition.

(A) qRT-PCR analysis showing relative *BMX* expression levels for tumor spheroids derived from SK-N-AS xenografts and transduced with either a *BMX*- (sh*BMX#1*) or GFP-targeting (shControl) shRNA sequence (mean ± SEM of three technical replicates is shown). **(B) and (C)** Decrease in viability (**B**, *left*), proliferation (**B**, *right*) and self-renewal **(C)** for spheroids derived from SK-N-AS xenografts, and transduced with sh*BMX*#1 lentivirus. Statistical analyses were performed using the unpaired t-test. Mean ± SEM values of three technical replicates are shown. Individual values are shown for **(C)**. **(D)** Representative micrographs depicting marked changes in spheroid morphology for the primary NB1 and NB4 models, as well as xenograft-derived SK-N-AS spheroids treated with BMX-IN-1 at 10μM or DMSO for four days. (Scale bars, 100 μm. Magnification, 10X). **(E)** Bar plots illustrating the decrease in viability for SK-N-AS spheroids as in **(D)**, treated with increasing concentrations of BMX-IN-1 for four days. **(F)** qRT-PCR assessment of relative *BMX* expression levels for primary Ewing and synovial sarcoma spheroid models, as compared to NB1 spheroids. Mean ± SEM values of three technical replicates are shown. **(G)** Bar plots showing the viability of primary Ewing (*left*) and synovial sarcoma (*right*) spheroids upon treatment with increasing concentrations of BMX-IN-1 for four days. Statistical analyses were performed using the unpaired t-test. ns, not statistically significant. **(H)** Micrographs illustrating representative images of Ewing (*top*) and synovial sarcoma (*bottom*) spheroids exposed to increasing concentrations of BMX-IN-1 or DMSO as in **(G)**. (Scale bars, 100 μm. Magnification, 10X). **(I)** Bar plots indicating no changes in the viability of adherent cells generated from NB1(*left*) and NB4(*right*) spheroids upon treatment for four days with increasing concentrations of BMX-IN-1 versus DMSO. (Scale bars, 300 μm. Magnification, 10X). **(J)** Micrographs illustrating representative images of NB1 (*top*) and NB4 (*bottom*) adherent cells exposed to 10 μm of BMX-IN-1 as in **(I)**. **(K)** Heatmap showing the mean RPKM values of the potential low-affinity targets of BMX-IN-1 in NB1 and NB4 spheroids and matched adherent models from the RNAseq data shown in **Fig. 2A**. **(L)** Immunoblotting for the detection of pSTAT3-Tyr705 and total STAT3 proteins in NB1 (*left panel*) and NB4 (*right panel*) spheroids following treatment with the BMX inhibitor BMX-IN-1 (10 μM) for three days, as compared to DMSO-treated cells.

Supplementary Figure 3

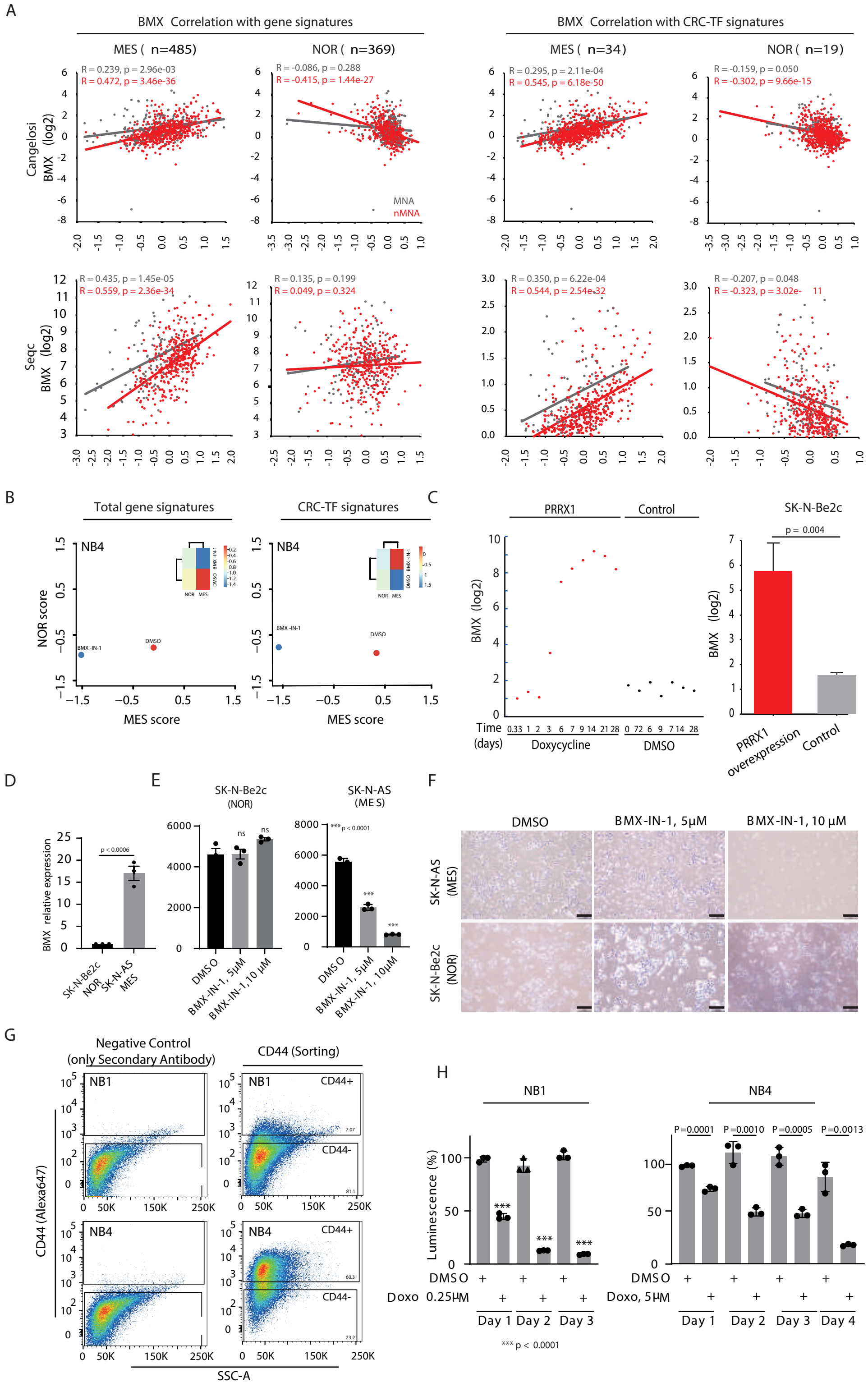

Supplementary Figure 3: BMX is involved in the maintenance of the MES phenotype of NB tumor cells.

(A) Scattered plots showing the correlation between *BMX* expression and the total MES (n=485) or NOR (n=369) gene signature *(left panels)* or the CRC-TFs of the MES (n=34) or NOR (n=19) cellular states (*right panels*) in primary tumors from the Cangeloshi and Seqc cohorts (R2 genomics platform). **(B)** Scatter plot and heatmap showing the marked changes in cell phenotype upon three days of 10µM BMX-IN-1 treatment for NB4 spheroids versus DMSO-treated control. Scores calculated using the total *(left panel)* or the restricted CRC-TFs (r*ight panel)* MES and NOR gene signatures used in (**A**). **(C)** Dot plot (*left*) and bar graph (*right*) depicting the changes in *BMX* expression for the SK-N-Be2c cell line engineered for the doxycycline-inducible expression of the PRRX1 TF. The data is extracted from the Versteeg - 17 dataset available at R2 genomics platform. (**D**) Bar plots showing the differences in BMX transcript levels between SK-N-Be2c and SK-N-AS adherent cells. Mean ± SEM values of three technical replicates of qRT-PCR are shown. (**E**) Bar graphs showing the changes in viability of SK-N-Be2c and SK-N-AS adherent cells upon treatment with increasing concentrations of BMX-IN-1 for 4 days. (**F**) Representative images showing the effect of 5 or 10µM BMX-IN-1 treatment on SK-N-AS (*top panels*) and SK-N-Be2c *(bottom panels)* adherent cells after 4 days of exposure versus DMSO. Scale bars, 100 µm. Magnification, 10X. (**G**) FACS dot plots showing the gating parameters used to sort CD44-positive and -negative NB1 (*top*) and NB4 (*bottom*) spheroids cell populations as in **Figure 6C**. Cells stained with only the secondary antibody were used as negative control. **(H)** Bar graphs illustrating the time course changes in spheroid viability following treatment with Doxorubicin. NB1 spheroids were treated with 0.25µM Doxorubicin (*left panel*), while NB4 spheroids were treated with 5µM Doxorubicin (*right panel*). Mean values ± SEM from three technical experiments are depicted. Statistical analyses were conducted using the unpaired t-test.
