# Supplementary material for: Preclinical spheroid models identify BMX as a therapeutic target for metastatic MYCN nonamplified neuroblastoma
Source: JCI Insight. 2024 Jul 22;9(14):e169647. doi: 10.1172/jci.insight.169647 (PMC11383371; doi:10.1172/jci.insight.169647)

Full unedited gel for Supplementary Figure S2L, Left panel

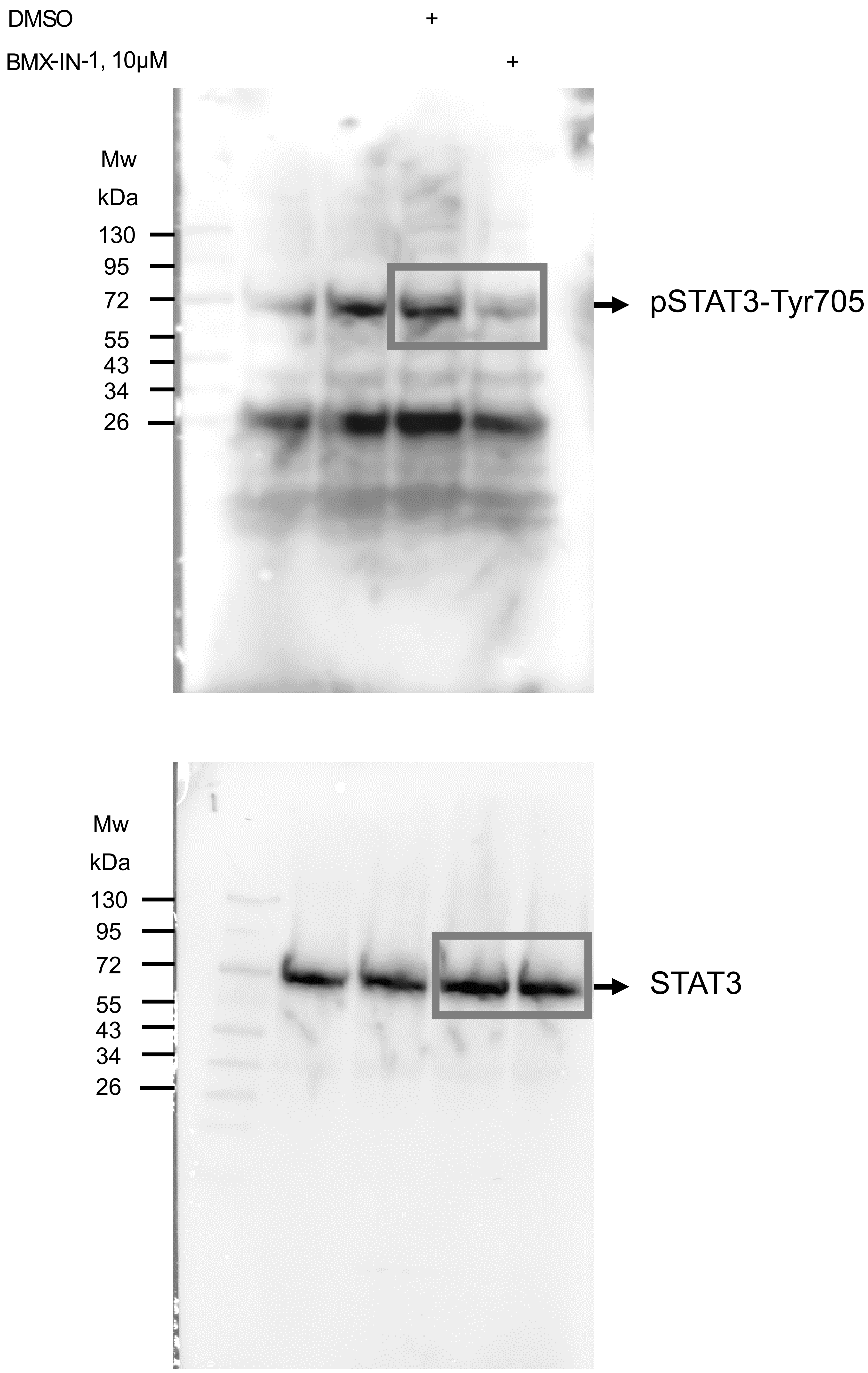

Full unedited gel for Supplementary Figure S2L, Right panel

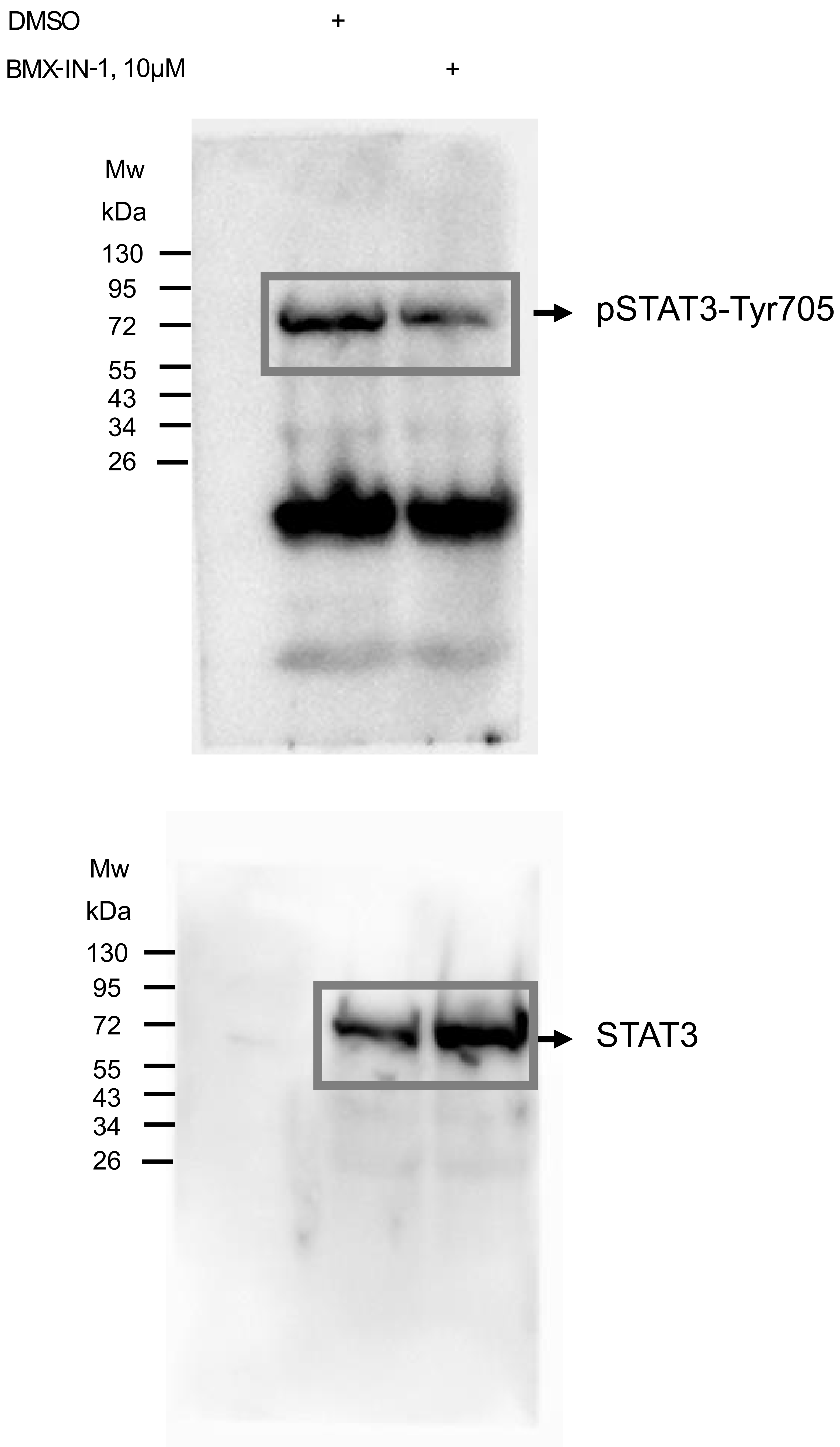

Supplement: Unedited blot and gel images [file jciinsight-9-169647-s201.pdf]
